# The Meckel-Gruber syndrome protein TMEM67 controls basal body positioning and epithelial branching morphogenesis in mice via the non-canonical Wnt pathway

**DOI:** 10.1242/dmm.019083

**Published:** 2015-06-01

**Authors:** Zakia A. Abdelhamed, Subaashini Natarajan, Gabrielle Wheway, Christopher F. Inglehearn, Carmel Toomes, Colin A. Johnson, Daniel J. Jagger

**Affiliations:** ^1^ Ciliopathy Research Group, Section of Ophthalmology and Neurosciences, Leeds Institute of Molecular Medicine, University of Leeds, Leeds LS9 7TF, UK; ^2^ Department of Anatomy and Embryology, Faculty of Medicine, Al-Azhar University, Cairo 11844, Egypt; ^3^ UCL Ear Institute, University College London, 332 Gray's Inn Road, London WC1X 8EE, UK

**Keywords:** TMEM67, Meckelin, MKS3, Wnt signalling, Planar cell polarity, PCP, Stereocilia, Kinocilia, Primary cilia, Hair bundle, Ciliopathy

## Abstract

Ciliopathies are a group of developmental disorders that manifest with multi-organ anomalies. Mutations in *TMEM67* (*MKS3*) cause a range of human ciliopathies, including Meckel-Gruber and Joubert syndromes. In this study we describe multi-organ developmental abnormalities in the *Tmem67^tm1Dgen/H1^* knockout mouse that closely resemble those seen in *Wnt5a* and *Ror2* knockout mice. These include pulmonary hypoplasia, ventricular septal defects, shortening of the body longitudinal axis, limb abnormalities, and cochlear hair cell stereociliary bundle orientation and basal body/kinocilium positioning defects. The basal body/kinocilium complex was often uncoupled from the hair bundle, suggesting aberrant basal body migration, although planar cell polarity and apical planar asymmetry in the organ of Corti were normal. TMEM67 (meckelin) is essential for phosphorylation of the non-canonical Wnt receptor ROR2 (receptor-tyrosine-kinase-like orphan receptor 2) upon stimulation with Wnt5a-conditioned medium. ROR2 also colocalises and interacts with TMEM67 at the ciliary transition zone. Additionally, the extracellular N-terminal domain of TMEM67 preferentially binds to Wnt5a in an *in vitro* binding assay. Cultured lungs of *Tmem67* mutant mice failed to respond to stimulation of epithelial branching morphogenesis by Wnt5a. Wnt5a also inhibited both the Shh and canonical Wnt/β-catenin signalling pathways in wild-type embryonic lung. Pulmonary hypoplasia phenotypes, including loss of correct epithelial branching morphogenesis and cell polarity, were rescued by stimulating the non-canonical Wnt pathway downstream of the Wnt5a-TMEM67-ROR2 axis by activating RhoA. We propose that TMEM67 is a receptor that has a main role in non-canonical Wnt signalling, mediated by Wnt5a and ROR2, and normally represses Shh signalling. Downstream therapeutic targeting of the Wnt5a-TMEM67-ROR2 axis might, therefore, reduce or prevent pulmonary hypoplasia in ciliopathies and other congenital conditions.

## Abstract (precis)

**Highlighted Article:** TMEM67 is a receptor of non-canonical Wnt signalling, implicating the Wnt5a-TMEM67-ROR2 axis during developmental signalling and disruption in ciliopathy disease state.

## INTRODUCTION

Primary cilia are microtubule-based organelles that sense and transduce extracellular signals on many mammalian cell types. The cilium is known to have essential roles throughout development in mechanosensation (Praetorius and Spring, 2001; Nauli et al., 2003), signal transduction through the Hedgehog-, Wnt- and PDGFRα-signalling pathways (Huangfu et al., 2003; Simons et al., 2005; Schneider et al., 2005) and in the establishment of left-right asymmetry (Nonaka et al., 1998). Primary cilia have a complex ultrastructure with a compartmentalisation of molecular components that combine functional modules. Components that are required for both the formation and function of the cilium have to be transported from the cytoplasm of the cell by the process of intraflagellar transport (IFT). Mutations in proteins that are structural or functional components of the primary cilium cause a group of human inherited conditions known as ciliopathies (Adams et al., 2008). The loss of these components can disrupt ciliary functions, such as the control of protein entry and exit from the cilium, the possible trafficking of essential ciliary components, and the regulation of signalling cascades and control of the cell cycle. Many proteins that are mutated in ciliopathies are localised to the transition zone, a compartment of the proximal region of the cilium (Szymanska and Johnson, 2012; Reiter et al., 2012). In particular, a protein complex at the transition zone known as the ‘MKS-JBTS module’ contains many of the proteins mutated in Meckel-Gruber syndrome (MKS) and Joubert syndrome (JBTS) (Garcia-Gonzalo et al., 2011; Sang et al., 2011).

MKS is the most severe ciliopathy, and is a lethal-recessive neurodevelopmental condition. The central nervous system (CNS) defects often comprise occipital encephalocele, rhombic roof dysgenesis and prosencephalic dysgenesis. Cystic kidney dysplasia and hepatic developmental defects are essential diagnostic features of MKS and, although the CNS defects are considered to be obligatory features, they have a more variable presentation. Other occasional features include post-axial polydactyly, shortening and bowing of the long bones, retinal colobomata and situs defects. To date, mutations in eleven genes have been described as a cause of MKS. However, mutations in the *TMEM67/MKS3* gene are the most common cause of MKS, accounting for over 15% of all MKS cases in unselected cohorts (Khaddour et al., 2007; Consugar et al., 2007; Szymanska et al., 2012), with mutations in *TMEM67* associated frequently with a diagnosis of malformation of the ductal plate in the liver (Khaddour et al., 2007; Consugar et al., 2007; Szymanska et al., 2012). *TMEM67* encodes TMEM67 (transmembrane protein 67, also known as meckelin), a 995 amino-acid-long transmembrane protein with structural similarity to Frizzled receptors (Smith et al., 2006). TMEM67/meckelin (hereafter called TMEM67) contains an extracellular N-terminal domain with a highly conserved cysteine-rich repeat domain (CRD), a predicted β-pleated sheet region and seven predicted transmembrane regions (Abdelhamed et al., 2013). TMEM67 is a component of the MKS-JBTS module at the transition zone. This functional module includes other transmembrane proteins, namely the Tectonic proteins (TCTN1 to 3), TMEM17, TMEM231 and TMEM237, as well as C2-domain proteins (jouberin/AHI1 and CC2D2A) (Sang et al., 2011; Garcia-Gonzalo et al., 2011; Huang et al., 2011; Chih et al., 2011). Transition zone proteins are thought to form a diffusion barrier at the base of the cilium that restricts entrance and exit of both membrane and soluble proteins (Williams et al., 2011; Garcia-Gonzalo et al., 2011).

**TRANSLATIONAL IMPACT**

**Clinical issue**

Mutations in proteins that are structural or functional components of the primary cilium (a microtubule-based mechanosensor organelle present in many mammalian cells) cause a group of comparatively common human inherited conditions known as ciliopathies. Most clinical features of ciliopathies, such as renal cystic dysplasia, are well-described. However, pulmonary hypoplasia (a congenital malformation of the lungs) is a consistent finding in a perinatal lethal group of skeletal ciliopathies (the short rib polydactyly syndromes) and might be under-reported in another severe ciliopathy [Meckel-Gruber syndrome (MKS)], despite being considered as the leading cause of death in individuals with MKS.

**Results**

To determine a possible disease mechanism for pulmonary hypoplasia in ciliopathies, this study characterises the transmembrane protein 67 knockout (*Tmem67^−/−^*) mouse model of MKS and the function of the TMEM67 protein. Pulmonary hypoplasia is a nearly consistent finding in *Tmem67^−/−^* embryos and pups. The study shows that TMEM67 is a receptor of non-canonical Wnt signalling that preferentially binds Wnt5a and mediates downstream signalling through receptor tyrosine kinase-like orphan receptor 2 (ROR2) as a co-receptor. Previous data and the present study confirm that loss or mutation of any component in the Wnt5a-TMEM67-ROR2 axis contributes to the pulmonary hypoplasia, condensed mesenchyme and impaired development of the alveolar system observed in the ciliopathy disease state. Lung branching morphogenesis in *Tmem67^−/−^* *ex vivo*-cultured lungs is rescued by treatment with calpeptin, an activator of RhoA (a downstream effector of the non-canonical Wnt signalling pathway).

**Implications and future directions**

These results provide the first evidence that TMEM67 is a receptor, and implicate the Wnt5a-TMEM67-ROR2 axis during developmental signalling of many lung tissues. In particular, this study emphasises the importance of downstream effectors of non-canonical Wnt signalling during lung development, and the dysregulation of this pathway in the ciliopathy disease state. Targeting these effectors could, therefore, provide the potential basis for therapeutic intervention to reduce or prevent pulmonary hypoplasia in ciliopathies and, perhaps, other congenital conditions for which pulmonary hypoplasia is a complication.

Loss or dysfunction of cilia in MKS causes complex de-regulation of normal key pathways of embryonic development, such as Wnt and Shh signalling (Abdelhamed et al., 2013). In particular, primary cilia have been proposed to mediate a negative modulatory effect on the canonical Wnt/β-catenin pathway (Simons et al., 2005; Gerdes et al., 2007; Corbit et al., 2008; Lancaster et al., 2011). In contrast, less is known about the possible regulatory roles of cilia and ciliary compartments on the non-canonical pathways of Wnt signalling. Downstream effects of non-canonical Wnt signalling – also referred to as planar cell polarity (PCP) – result in cytoskeletal actin rearrangements that cause changes in cell morphology and their directed orientation relative to a planar axis within an epithelium. Actin cytoskeleton remodelling is mediated by Rho proteins, a family of small GTPases that regulate many aspects of intracellular actin dynamics. In vertebrates, PCP signalling is required for correct convergent extension (Jessen et al., 2002; Ybot-Gonzalez et al., 2007) that, when disrupted, can cause neural tube defects, misorientation of hair cells and disruption of stereociliary bundles in the mammalian cochlea (Montcouquiol et al., 2003), and misorientation of hair follicles in the epidermis (Devenport and Fuchs, 2008). The importance of cilia for PCP signalling has been shown for ciliary proteins (namely, certain Bbs proteins and Ift88) that are required for the correct regulation of basal body polarisation in the cochlea (Ross et al., 2005; Jones et al., 2008). Furthermore, the core PCP protein Dishevelled (Dvl) and other core PCP proteins (such as Dubroya, Frizzled, and Celsr2 and Celsr3) are involved in the assembly and remodelling of the actin cytoskeleton in apical cellular regions (Oishi et al., 2006; Valente et al., 2010; Tissir et al., 2010), allowing subsequent ciliogenesis by the docking basal bodies to the apical cellular membrane (Park et al., 2008). Consistent with a role in non-canonical Wnt signalling, TMEM67 is required for centriolar migration to the apical membrane (Dawe et al., 2007), as well as the regulation of actin cytoskeleton remodelling and RhoA activity (Dawe et al., 2009). Furthermore, Wnt5a (which activates the non-canonical but inhibits the canonical Wnt pathway) stimulated the aberrant formation of extensive actin stress fibres in the absence of TMEM67 (Abdelhamed et al., 2013). However, the role of TMEM67 in non-canonical Wnt signalling or the PCP signalling system is unknown, and it remains undetermined whether TMEM67 binds to the Wnt5a ligand or is essential for co-receptor function.

To begin to answer these questions, the present study focuses on PCP and non-canonical Wnt signalling defects in the recently characterised *Tmem67^tm1Dgen/H^* knockout mouse (Abdelhamed et al., 2013; Garcia-Gonzalo et al., 2011), hereafter referred to as the *Tmem67^−/−^* knockout mutant. We now show that the pulmonary and cardiological phenotypes of *Tmem67^−/−^* mutant embryos closely recapitulate those of *Wnt5a* and *Ror2* mutant mice (Oishi et al., 2003). To substantiate a possible role of TMEM67 in the non-canonical Wnt signalling pathway, we examined the morphogenesis of the cochlea in neonatal *Tmem67^−/−^* mice, a well-characterised model system to determine PCP defects in a developing embryo (Jones and Chen, 2007). Analysis of the orientation of stereociliary hair bundles, and the positioning of primary cilia and basal bodies, demonstrated a consistent TMEM67-dependent effect on cochlear PCP. We then used biochemical methods to show the domains of interaction between TMEM67 and either Wnt5a or the non-canonical Wnt receptor ROR2 (receptor-tyrosine-kinase-like orphan receptor 2). We also functionally characterised the response of lung tissue explanted *ex vivo* for external Wnt5a stimulation, showing that normal epithelial branching morphogenesis and cell polarity was lost in the absence of TMEM67 but could be rescued by activation of RhoA. Our results suggest that TMEM67 has a putative receptor/co-receptor function in non-canonical Wnt signalling, preferentially binding Wnt5a with the extracellular cysteine-rich domain (CRD) and mediating downstream signalling through ROR2 as a co-receptor. TMEM67 might, therefore, be essential for ROR2 function and the correct activation of downstream non-canonical Wnt signalling cascades.

## RESULTS

### *Tmem67^−/−^* embryos recapitulate the phenotypes of *Wnt5a* and *Ror2* knockout animals

**Fig. 1.**
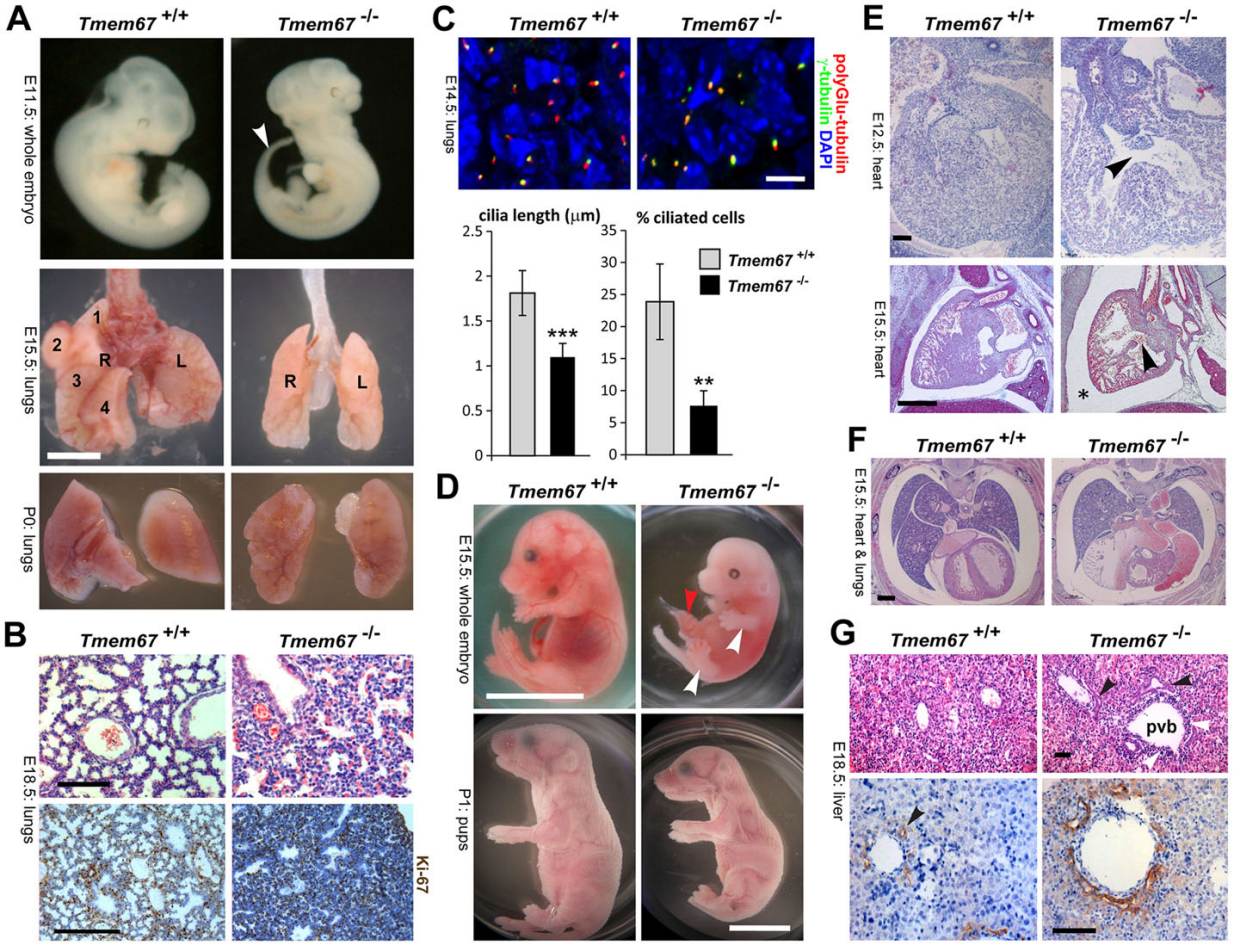
**Gross anatomical malformations, laterality defects, cardiac defects and pulmonary hypoplasia in *Tmem67^−/−^* mutant mouse embryos and pups.** (A) Upper panels: whole-mount E11.5 embryos showing the earliest sign of laterality defects with inverted tail turning (arrowhead) in a *Tmem67^−/−^* mutant embryo. Whole-mount lungs of E15.5 embryos (middle panels) and P0 pups (lower panels). *Tmem67^−/−^* E15.5 mutant embryos had identical left (L) and right (R) lungs, indicating left lung isomerism. Lobes of the right lung in *Tmem67^+/+^* are numbered as indicated. Scale bar: 1500 μm. (B) Upper panels: H&E-stained lung tissue section showing pulmonary hypoplasia, congested vessels and delayed development of the pulmonary alveoli in an E18.5 *Tmem67^−/−^* embryo. Lower panels: immunohistochemical staining for Ki-67 in E18.5 lung sections. Scale bars: 40 μm. (C) IF microscopy of E14.5 lung-tissue sections stained for primary cilia (acetylated α-tubulin; red), basal bodies (γ-tubulin; green) and for nuclei with DAPI (blue). Scale bar: 10 μm. Bar graphs show primary cilia length and number in *Tmem67^+/+^* and *Tmem67^−/−^* tissues. Statistical significance of the pairwise comparison are **P<0.01 and ***P<0.001 (Student's two-tailed *t*-test). Error bars indicate s.e.m. (D) Upper panels: whole-mount E15.5 embryo images showing generally delayed development, underdeveloped limbs (white arrowheads) and omphalocele (red arrowhead) in *Tmem67^−/−^* embryos, with details of limb dysplasia shown below. Lower panels: whole-mount P1 pups, showing reduced body longitudinal axis in the *Tmem67^−/−^* pups. Scale bars: 1 cm. (E) Upper panels: H&E-stained horizontal section through the chest cavity of E12.5 *Tmem67^+/+^* and *Tmem67^−/−^* animals showing a ventricular septal defect (VSD) (arrowhead) in the mutant. Scale bars: 100 μm. Lower panels: VSD (arrowhead) in an E15.5 sagittal heart section. Scale bar: 200 μm. (F) Horizontal sections through the thoracic cavity of the *Tmem67^−/−^* mutant and wild-type control showing aberrant lung lobulation, dextrocardia, major cardiac malformation and cardiac oedema or pericardial effusion (asterisk) in the *Tmem67^−/−^* embryo. Scale bars: 100 μm. (G) H&E- (upper panels) and IHC- (lower panels) stained E18.5 liver-tissue sections. H&E sections show a persistent double-layered ductal plate (black arrowheads) around the portal vein branches (pvb) and abnormally accumulating cells around the pvb in *Tmem67^−/−^* embryos (white arrowheads). IHC-stained liver sections for cytokeratin-19 show a double-layered ductal plate and multiple bile ducts in *Tmem67^−/−^* embryos. A normal bile duct in the *Tmem67^+/+^* section is indicated (arrowhead). Scale bars: 50 μm.

The majority of mutant *Tmem67^−/−^* pups died at birth, and none lived beyond the second postnatal day (P1), most probably because of pulmonary hypoplasia and complex cardiac malformations that include ventricular septal defect (VSD). Both phenotypes were consistent with anomalies detected in *Wnt5a* and *Ror2* mutant animals. Morphological and histological examination of *Tmem67* mutants showed that the lungs were hypoplastic (Fig. 1A) with failure of the pulmonary alveoli to develop (Fig. 1B,C). Interstitial cells also showed increased cell proliferation as determined by staining for the proliferation marker Ki-67 (Fig. 1B). Primary cilia were significantly reduced in both length, and number on cells forming the pulmonary alveoli and distal air sacs in *Tmem67^−/−^* embryonic lungs (Fig. 1C).

Limb dysplasia, omphalocele and intrauterine growth retardation were detected in 20% (*n*=4/20) of *Tmem67^−/−^* embryos (Fig. 1D). Caudal truncation with a shortened anterior-posterior axis was detected in 60% of mutant pups (*n*=12/20) (Fig. 1D). A small proportion of E11.5 *Tmem67^−/−^* embryos (*n*=1/12) developed an inverted tail turning (Fig. 1A), the earliest sign of laterality defects. Later in development at the perinatal (E15.5) and early postnatal stages (P0), 100% (*n*=7/7) of investigated mutant animals had left pulmonary isomerism (Fig. 1A). Both the right and left lungs appeared indistinguishable from each other and were formed of two identical symmetrical lung lobes. In the *Tmem67^+/+^* wild-type embryos, the right and left lungs were easily differentiated by the identification of four and one lobes, respectively (Fig. 1A).

Cardiac oedema consistently developed in most of the animals analysed. Complex cardiac developmental defects, including ventricular septal defect, atrial septal defect and dextrocardia, were common malformations detected in *Tmem67^−/−^* embryos (*n*=6/8) (Fig. 1E,F). All mutant *Tmem67^−/−^* embryos showed evidence of a ductal plate malformation and the retention of multiple primitive bile duct structures (Fig. 1G), consistent with the hepatic developmental anomalies observed in human patients carrying mutations of *TMEM67* (Adams et al., 2008; Khaddour et al., 2007; Consugar et al., 2007; Szymanska et al., 2012), and in *Wnt5a* and *Ror2* mutant mice (Kiyohashi et al., 2013). The pulmonary, cardiological and hepatic phenotypes of *Tmem67^−/−^* mutant embryos, therefore, closely recapitulate those of *Wnt5a* and *Ror2* mutant mice (Oishi et al., 2003). In addition, the caudal truncation and shortened anterior-posterior axis in P0 *Tmem67^−/−^* mutant pups is similar to that of *Wnt5a* knockout mice.

### Cochleae of neonatal *Tmem67^−/−^* mutants display abnormalities of hair-bundle orientation with uncoupling of primary cilia and basal bodies, but have normal PCP and apical planar asymmetry

**Fig. 2.**
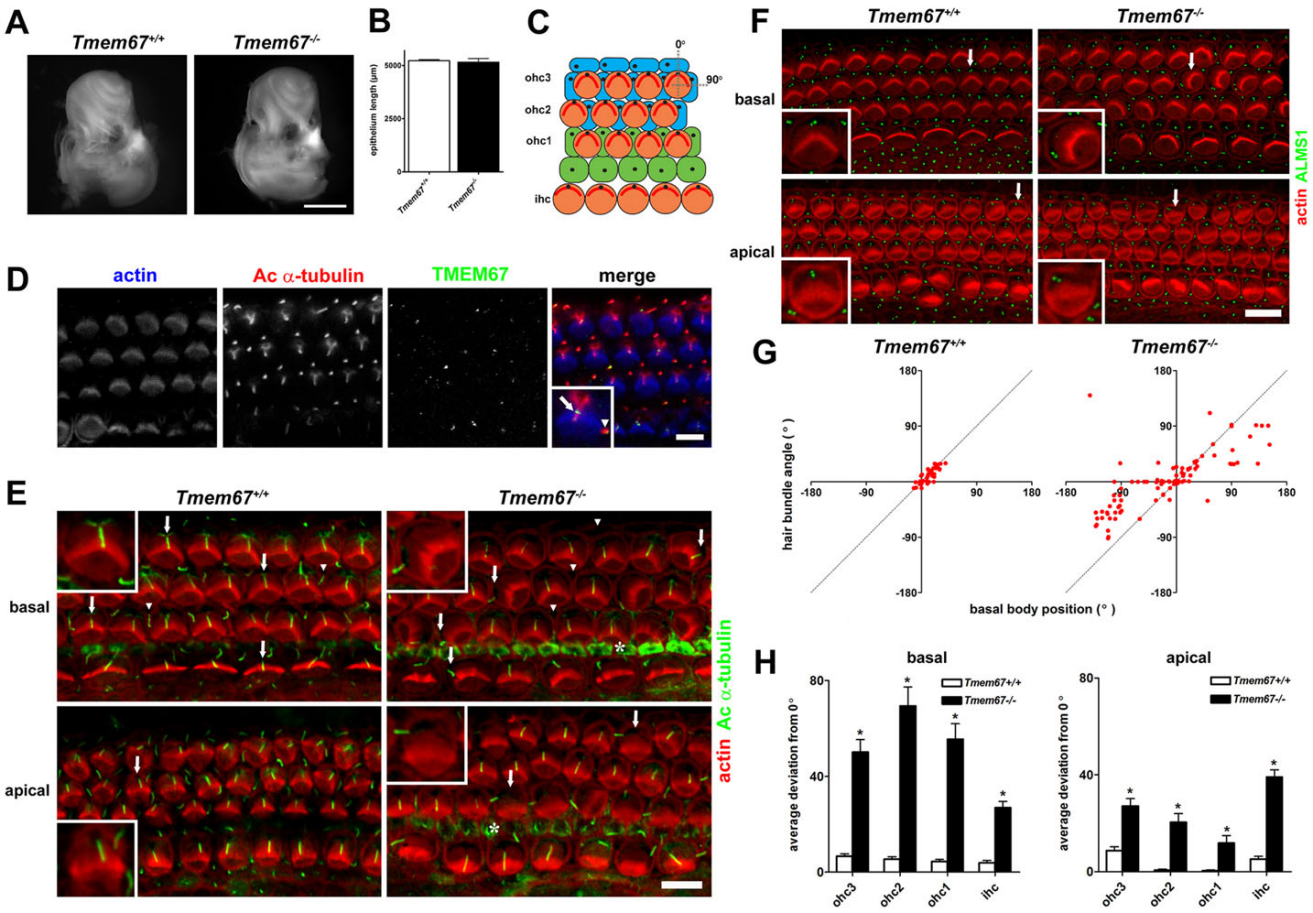
**Orientation defects in stereociliary hair bundles with uncoupling from kinocilium and basal body position of hair cells in the organ of Corti of neonatal *Tmem67^−/−^* mice.** (A) Cochleae dissected from P0 *Tmem67^+/+^* mice (control, left) were indistinguishable from those of *Tmem67^−/−^* littermates (right). Scale bar: 1 mm. (B) Total length measurements of phalloidin-stained organ of Corti were not significantly different between control and mutant animals (*n*=4 cochleae per genotype). (C) Schematic representation of cellular architecture of the neonatal organ of Corti. There is a single row of inner hair cells (ihc) located at the neural edge of the sensory epithelium, and three rows of outer hair cells (ohc1-3) spanning the abneural portion. The hair cell stereociliary bundles (red) are regularly oriented, with their vertices pointing towards the abneural pole, corresponding to an alignment of 0° (denoted by vertical dotted line). A line of alignment to 90° is also shown for reference. Ohc are surrounded by a mosaic of non-sensory supporting cells, including pillar cells (green) and Deiters' cells (blue). Primary cilia are represented as black dots. (D) Confocal projections of P0 *Tmem67^+/+^* organ of Corti mid-turn region (50% of cochlear length) stained for actin using phalloidin to demarcate stereociliary hair bundles (blue), acetylated α-tubulin antibody (cilia; red) and TMEM67 (green). TMEM67 decorates the proximal regions of cilia in both hair-cell types and supporting cells. The magnified inset shows TMEM67 ciliary localisation in a single outer hair cell (arrow) and an adjacent Deiters' cell (arrowhead). Scale bar: 10 µm. (E) On the surface of the basal turn (10-20% of cochlear length) in the organ of Corti of a P0 *Tmem67^+/+^* mouse (left), there was a regular arrangement of V-shaped stereociliary ohc hair bundles (phalloidin; red), with kinocilia (acetylated α-tubulin; green) positioned at the abneural pole (around 0°) of hair cells in all three rows (arrows; shown in magnified insets). Each kinocilium was in close apposition to the vertex of each hair bundle. Non-sensory supporting cells were also ciliated (arrowheads). In a *Tmem67^−/−^* littermate (right) kinocilia were often mislocalised from the abneural pole of the hair cell (arrows; shown in magnified insets), and in these cells the orientation of the hair bundle was uncoupled from the kinocilium position. Adjacent supporting cells were often not ciliated (arrowheads). Similar effects were seen in the apical turn region (∼70-80% cochlear length). Cytoskeletal staining of inner pillar cells is indicted by asterisks. Scale bar: 10 µm. (F) Basal body position and hair-bundle orientation were tightly coupled in basal and apical regions of the *Tmem67^+/+^* organ of Corti (left). Uncoupling of hair-bundle orientation from basal body position was apparent in all rows of hair cells, in both basal and apical regions in *Tmem67^−/−^* cochleae (detail indicated by arrows is shown in magnified insets). Scale bar: 10 µm. (G) Scatter plots showing hair-bundle orientation versus position of the basal body for individual ohc in the basal region (corresponding to ∼10-20% of cochlear length) of a *Tmem67^+/+^* mouse (left; *n*=230) and a *Tmem67^−/−^* littermate (right; *n*=165). Dashed lines indicate the position of perfect correlation (Pearson's coefficient of correlation, *r*=1). (H) Genotype-specific differences in basal body position for individual hair cell rows in basal (10-20%, left) and apical (70-80%, right) cochlear regions. Average deviations from 0° were significantly different between the genotypes for all rows (pairwise comparisons are *P<0.001; Student's unpaired *t*-test) in both basal and apical regions. Error bars indicate s.e.m.

To further investigate the possible role of TMEM67 in the non-canonical Wnt signalling pathway, we examined the morphogenesis of the cochlea in neonatal *Tmem67^−/−^* mice. We mapped the distribution of TMEM67 in the neonatal organ of Corti, and analysed the orientation of stereociliary hair bundles and the position of primary cilia to determine TMEM67-dependent effects on cochlear PCP. Cochleae from P0 *Tmem67^−/−^* mice were normal in appearance and comparable in size to those of littermate controls (Fig. 2A). Phalloidin staining of whole-mount preparations of the organ of Corti (the sensory neuroepithelium) revealed that the total epithelial length was not different between the genotypes (Fig. 2B), suggesting that TMEM67 does not play a direct role in the PCP-associated convergent extension mechanisms that underlie growth of the organ of Corti along the baso-apical axis (Dabdoub and Kelley, 2005). The organ of Corti, which is shown in schematic form in Fig. 2C, is an epithelial mosaic comprising a single row of inner hair cells (ihc) and generally three rows of outer hair cells (ohc), which are interspersed with non-sensory supporting cells. During normal development, all cells in the epithelium possess a single cilium that projects from their apical (luminal) surface, whereas hair cells can be identified by their actin-containing stereociliary bundles. TMEM67 was localised to the proximal regions of acetylated α-tubulin-stained cilia of hair cells and the supporting cells of P0 wild-type mice (Fig. 2D), consistent with its previously described localisation to the ciliary transition zone (Simons et al., 2005; Garcia-Gonzalo et al., 2011).

Along the whole baso-apical axis of both *Tmem67^+/+^* and *Tmem67^−/−^* cochleae there was a single continuous row of ihc located along the neural (medial) edge of the sensory epithelium (Fig. 2E). Similarly, there were three continuous rows of ohc running parallel to the abneural (lateral) edge in all animals. The normal cochlear morphogenesis further suggests that TMEM67 does not contribute to cochlear convergent extension. The phalloidin-stained hair bundles of *Tmem67^+/+^* ihc and ohc were all regularly oriented (Fig. 2E), with the vertex of the ‘V-shaped’ bundle generally directed towards 0° (the abneural pole; Fig. 2C). Similarly, the stereociliary hair bundles of ihc in neonatal *Tmem67^−/−^* mice had a regular orientation. However, there were marked abnormalities in the alignment of ohc stereociliary hair bundles in neonatal *Tmem67^−/−^* mice, a phenotype that was most noticeable in the basal cochlear turn, where ∼30% of place-matched ohc had misoriented bundles relative to the abneural pole. Misoriented ohc often retained a roughly V-shaped hair bundle (Fig. 2E; *Tmem67^−/−^* basal turn, inset). In the apical (least mature) regions, the ohc bundle abnormalities were still apparent but had a lower occurrence.

Primary cilia were detected on the surface of hair cells and non-sensory supporting cells in the basal cochlear region of *Tmem67^+/+^* mice (Fig. 2E). The primary cilia of hair cells (known as kinocilia) were all located close to the vertex of the regularly aligned hair bundles. Kinocilia were also detected on the surface of all *Tmem67^−/−^* hair cells, and these were located in approximately normal positions on ohc with hair bundles oriented towards 0°, and on some ihc. On ohc with noticeably misorientated hair bundles, the kinocilium was eccentrically localised and, consequently, found mispositioned relative to the bundle vertex. In such instances, the kinocilium rarely appeared to contact the tallest row of stereocilia at the rear of the bundle. In most ihc, although the hair bundle was oriented normally, kinocilia were positioned eccentrically and were not attached to the hair bundle. There was an absence of cilia on supporting cells in the lateral part of the organ of Corti of *Tmem67^−/−^* mutants, namely on the Deiters’ cells and outer pillar cells.

**Fig. 3.**
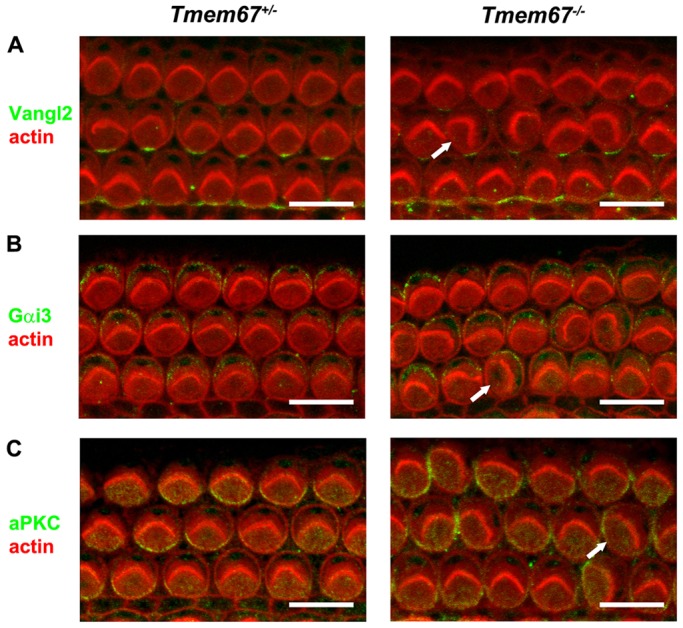
**Normal PCP and apical planar asymmetry in the organ of Corti of neonatal *Tmem67^−/−^* mice.** Confocal projections of P0 *Tmem67^+/−^* (left panels) and *Tmem67^−/−^* (right panels) basal turn organ of Corti (corresponding to 10-20% of cochlear length) stained for actin to demarcate stereociliary hair bundles and cell borders (red). (A) In both genotypes, Vangl2 (green) localised to supporting cells at the adherens junction with hair cells. (B) Gαi3 (green) is enriched in the lateral ‘bare zone’ on the apical surface of outer hair cells. (C) aPKC (green) is enriched in the medial/neural compartment on the apical surface of outer hair cells. Misaligned hair bundles in *Tmem67^−/−^* cochleae (arrows) are adjacent to normally expressed Vangl2, or display the normal asymmetric expression of Gαi3 and aPKC. Scale bars: 10 µm.

The uncoupling of cochlear cilia from hair bundles in neonatal *Tmem67^−/−^* mutants was further investigated by a quantitative analysis of the basal body position in ohc and ihc along the baso-apical axis of the organ of Corti (Fig. 2F,G), because the localisation of the basal body has been used as a measure of the PCP axis in hair cells (Jones and Chen, 2007). The basal body in hair cells could be delineated by the anti-ALMS1 antibody (Fig. 2F), allowing the precise measurement of position relative to 0°. Scatter plots of hair-bundle orientation versus basal-body position for individual basal-turn ohc demonstrated the variation of the uncoupling defect in *Tmem67* mutants (Fig. 2G). In *Tmem67^+/+^* hair cells there was close correlation between hair-bundle orientation and basal body position (Pearson's coefficient of correlation, *r*=0.86). For *Tmem67^−/−^* mutant ohc, although some cells had close coupling of the basal body and hair bundle, there was an overall broader distribution (*r*=0.71). An analysis of the average deviation of the basal body position from 0° (Fig. 2H) revealed significant mislocalisation in each row of *Tmem67^−/−^* hair cells along the mutant cochleae, and place-dependent variability within the medio-lateral axis. In contrast, the positional deviation of basal bodies in *Tmem67^+/+^* hair cells was identical to previous measurements of hair-bundle orientation at this gestational age (Jones and Chen, 2007). Distribution histograms for basal body position in hair cells (supplementary material Fig. S1) further demonstrated the variability of the mislocalisation along the baso-apical and medio-lateral axes of *Tmem67^−/−^* mutant cochleae. In contrast, both PCP and apical planar asymmetry were undisturbed in the organ of Corti of neonatal *Tmem67^−/−^* mice, by IF staining for the core PCP protein Vangl2 (Montcouquiol et al., 2003), and the asymmetrically localised GTP-binding protein alpha-i subunit 3 (Gαi3) and atypical protein kinase C (aPKC; Ezan et al., 2013) (Fig. 3).

### Basal body mislocalisation defects during hair cell differentiation in embryonic *Tmem67^−/−^* mutants

To further investigate the ontogeny of the basal body mispositioning in *Tmem67^−/−^* mutant hair cells during late gestation, we examined the sensory epithelium during a prenatal period, when hair cells and supporting cells begin to differentiate within the pro-sensory domain. The cell types can be distinguished first in the basal region between E14 and E15 in the mouse cochlea, and then along the whole baso-apical axis by E17 (Dabdoub and Kelley, 2005). At E15.5, hair cells can be clearly defined by phalloidin staining in the basal cochlear region (supplementary material Fig. S2). In the basal region, primary cilia were detected on *Tmem67^+/+^* hair cells and supporting cells (supplementary material Fig. S2A) but, as observed in P0 animals, *Tmem67^−/−^* supporting cells in the lateral region lacked primary cilia (supplementary material Fig. S2A). The kinocilium was localised centrally on the apical surface of a hair cell and, subsequently, migrated to the abneural pole (Jones et al., 2008). In the basal turn of E15.5 *Tmem67^+/+^* mice, ALMS1-labelled basal bodies had already migrated to the abneural pole in ihc and rows 1-2 of ohc (supplementary material Fig. S2B). In *Tmem67^−/−^* mutant littermates, ihc basal bodies appeared to have a largely normal localisation, but basal bodies of ohc in all rows were often found centrally or had apparently migrated eccentrically towards the cell periphery (supplementary material Fig. S2B,C). This suggests that TMEM67 regulates the migration of ohc basal bodies towards the cell periphery but not those of ihc, and might specify the final position of basal bodies in all hair cells relative to 0°. In the mid-turn region of both genotypes, ihc had polarised basal bodies but basal bodies in all ohc rows had a central localisation (supplementary material Fig. S2B,C), suggesting migration had yet to commence at this less developed region of the baso-apical axis.

### TMEM67 is required for negative regulation of the canonical Wnt/β-catenin signalling pathway by Wnt5a and interacts with ROR2

**Fig. 4.**
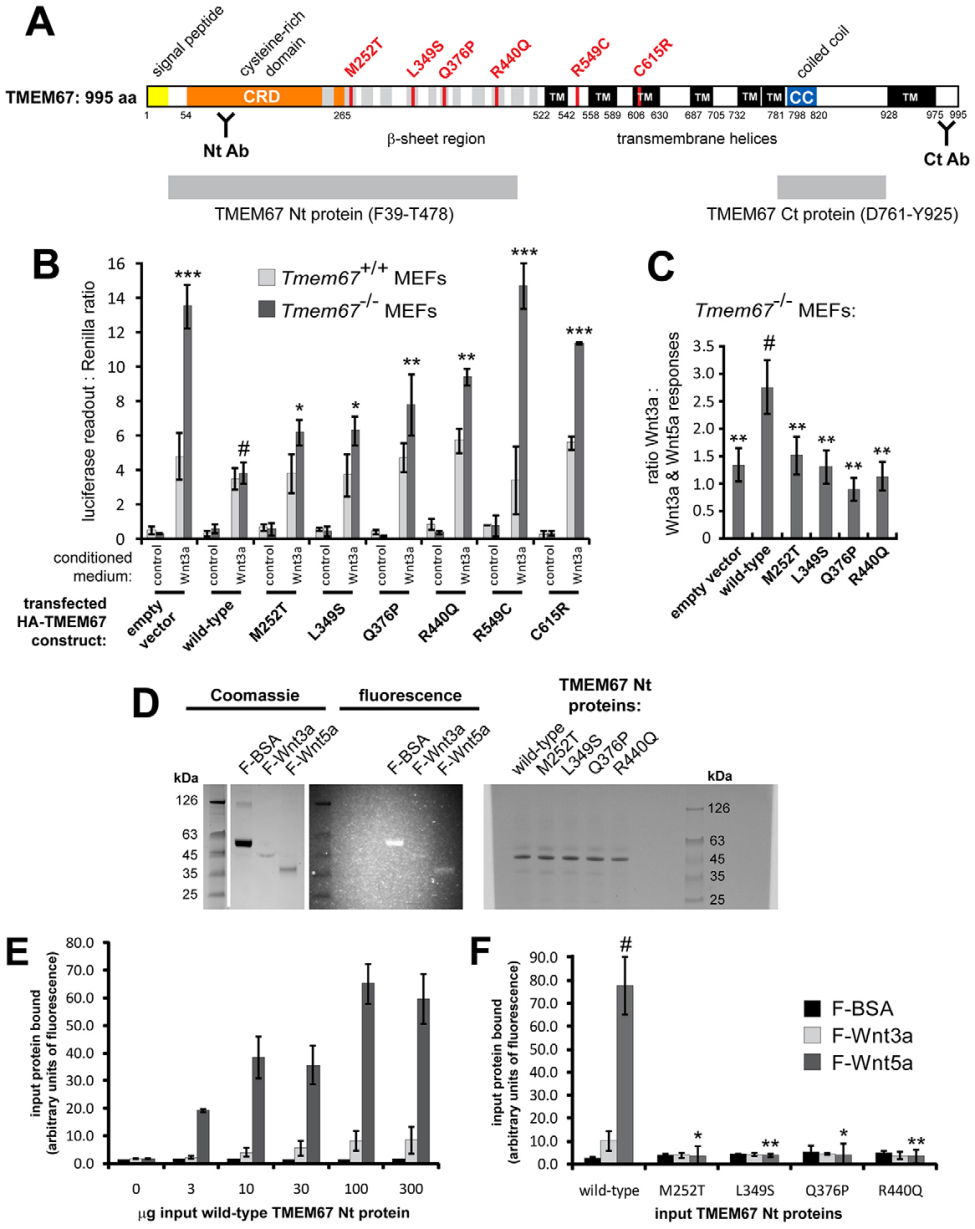
**Non-canonical Wnt signalling defects in *Tmem67^−/−^* cells and interaction of Wnt5a with the TMEM67 N-terminal domain.** (A) Schematic diagram of conserved domains and structural motifs within the TMEM67 protein, comprising a signal peptide (yellow), a cysteine-rich domain (CRD, orange), regions of β-sheet periodicity (grey), seven predicted transmembrane helices (TM, black) and a coiled-coil domain (CC, blue). Locations are indicated by amino acid residue (aa), with pathogenic missense mutations highlighted in red. The approximate locations of the two epitopes used to raise N-terminal (Nt) and C-terminal (Ct) rabbit polyclonal antibodies (Ab) are indicated. The TMEM67 regions used for exogenous protein expression are indicated by grey boxes. (B) TOPFlash assays to quantify canonical Wnt signalling activity in *Tmem67^+/+^* and *Tmem67^−/−^* MEFs, following treatment with either control L-cell or Wnt3a-conditioned media, as indicated, and co-transfection with empty vector control, wild-type HA-TMEM67, or HA-TMEM67 containing a series of pathogenic missense mutations. Wild-type HA-TMEM67 rescued de-regulated canonical Wnt signalling in *Tmem67^−/−^* cells, but missense constructs did not. (C) *Tmem67^−/−^* cells had a defective response to Wnt5a, expressed as the ratio of Wnt3a response:combined response to both Wnt3a and Wnt5a. The correct response to Wnt5a was only rescued with wild-type HA-TMEM67. Values shown are means of at least four independent replicates and error bars indicate ±s.e.m. The statistical significance of the pair-wise comparisons with wild-type HA-TMEM67 values (#) are represented as *P<0.05, **P<0.01 and ***P<0.001, Student's two-tailed *t*-test. (D) Left panel: Coomassie-stained SDS-PAGE analysis of fluorescence-labelled BSA (F-BSA), Wnt3a (F-Wnt3a) and Wnt5a (F-Wnt5a) proteins. Molecular masses of protein size standards (kDa) are indicated. Middle panel: the same gel photographed under UV light to show fluorescent labelling of BSA control, Wnt3a and Wnt5a proteins. Right panel: expression of TMEM67-Nt proteins (predicted molecular mass 48 kDa), containing the indicated missense mutations. (E) Preferential *in vitro* interaction of F-Wnt5a, but not F-Wnt3a or F-BSA negative control, with increasing amount of wild-type TMEM67-Nt. (F) Interaction of F-Wnt5a with wild-type TMEM67-Nt only, but not TMEM-Nt proteins containing the indicated missense mutations. Values shown are the means of three independent replicates and error bars indicate ±s.e.m. The statistical significance of the pair-wise comparisons with wild-type TMEM67-Nt values (#) are represented as *P<0.05 and **P<0.01, Student's two-tailed *t*-test.

We next used biochemical methods to substantiate that *Tmem67^−/−^* cells have a defect in the regulation of non-canonical Wnt signalling that is concomitant with loss of negative modulation of the canonical Wnt/β-catenin pathway. TMEM67 is a putative orphan receptor with similarities to the Frizzled proteins (Fig. 4A) (Smith et al., 2006; Abdelhamed et al., 2013), and we, therefore, next used the TOPFlash assay to quantify the ability of *Tmem67^+/+^* and *Tmem67^−/−^* mouse embryonic fibroblasts (MEFs) to respond to Wnt ligands. After co-transfection of the TOPFlash reporter constructs, treatment with Wnt3a stimulated basal levels of Wnt/β-catenin signalling by about fivefold in *Tmem67^+/+^* MEFs, but by 13.8-fold in mutant cells (Fig. 4B). Co-transfection with a wild-type TMEM67 construct completely rescued the normal response in *Tmem67^−/−^* MEFs by suppressing the deregulated canonical Wnt/β-catenin signalling responses to Wnt3a (Fig. 4B). However, TMEM67 constructs with the pathogenic missense mutations M252T, L349S, Q376P and R440Q in the extracellular N-terminal (Nt) domain of TMEM67 (Fig. 4A) were unable to restore normal basal levels of canonical Wnt/β-catenin signalling (Fig. 4B). Two other pathogenic missense mutations, R549C and C615R, located close to transmembrane helices (Fig. 4A), also did not rescue basal responses to Wnt3a (Fig. 4B). Although Wnt5a on its own had no effect on the canonical pathway (Abdelhamed et al., 2013), treating cells with a mixture of Wnt3a and Wnt5a showed that the latter ligand was able to competitively inhibit the Wnt3a response in wild-type cells, but only partially inhibited the Wnt3a response in mutant cells. In *Tmem67^−/−^* cells, the missense mutations in the extracellular Nt domain of TMEM67 did not rescue the competitive inhibition of Wnt3a canonical responses by Wnt5a (Fig. 4C). Wild-type *TMEM67* partially rescued the correct response – as expected (Fig. 4C), implying that Wnt5a modulates a non-canonical Wnt signalling response through TMEM67.

Since the cardiological and pulmonary phenotypes of *Tmem67^−/−^* mutant embryos (Fig. 1A-C,E,F) closely recapitulate those of *Wnt5a* and *Ror2* mutant mice, and because P0 pups exhibit a shortened anterior-posterior axis (Fig. 1D) similar to *Wnt5a* knockout mice, we hypothesised that TMEM67 is a potential receptor that directly binds Wnt ligands. To test this, we performed an *in vitro* binding assay using purified, fluorescein-labelled Wnt3a or Wnt5a proteins (Fig. 4D). Titration with increasing amounts of wild-type TMEM67-Nt protein (Fig. 4D), demonstrated a preferential binding to Wnt5a compared with Wnt3a (Fig. 4E). Missense mutations (M252T, L349S, Q376P and R440Q) in the extracellular N-terminal region of TMEM67 (Fig. 4A) completely abolished binding to Wnt5a (Fig. 4F). We were, however, unable to test the TMEM67-Nt R549C and C615R proteins because the proximity of hydrophobic residues in the transmembrane helices prevented efficient protein expression (data not shown).

**Fig. 5.**
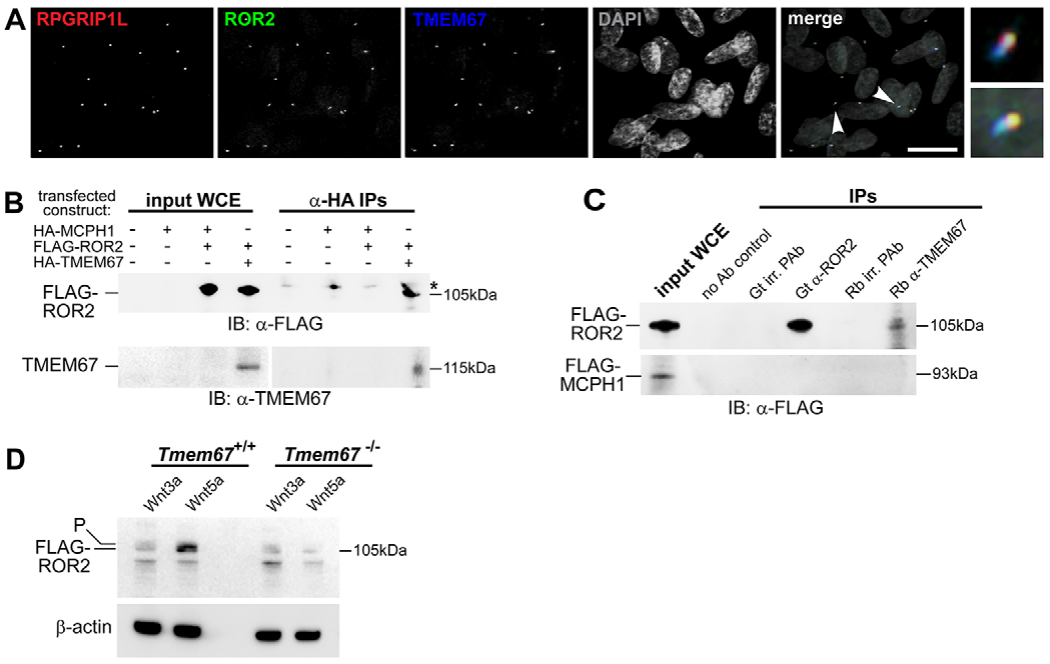
**The receptor tyrosine kinase-like orphan receptor ROR2 colocalises and interacts with TMEM67, and is dependent on this interaction for phosphorylation.** (A) Four-colour IF imaging showing that endogenous ROR2 (green) colocalizes with TMEM67 (blue) and RPGRIP1L (red) at the ciliary transition zone. Arrowheads indicate regions shown in magnified insets. DAPI is pseudocoloured in grey. Scale bar: 10 μm. (B) Anti-HA co-immunoprecipitations (IPs) demonstrating interaction between full-length exogenous HA-tagged TMEM67 (size 115 kDa) and FLAG-tagged ROR2 (size 105 kDa). Input whole-cell extracts (WCE) for the indicated transfected constructs are on the left. IP of an irrelevant protein (HA-tagged MCPH1) was a negative control. Results are shown for immunoblotting (IB) for anti-FLAG (upper panel) and anti-TMEM67 (lower panel). * indicates a non-specific band in IPs; see supplementary material Fig. S6 for full unprocessed images. (C) Upper panel: IPs demonstrating interaction between FLAG-tagged ROR2 and endogenous TMEM67. Input WCE is shown on the left, and negative control IPs include a no antibody (Ab) control and goat (Gt) and rabbit (Rb) irrelevant (irr.) polyclonal antibodies (PAb). Immunoblotting (IB) for anti-FLAG shows pulldown of FLAG-ROR2 by Gt anti-ROR2 and Rb anti-TMEM67. Lower panel: IPs with irrelevant protein (FLAG-MCPH1, size 93 kDa). (E) Loss of the active phosphorylated ROR2 isoform (labelled P) in mutant *Tmem67^−/−^* cells following Wnt5a treatment, compared with strong induction of the active isoform (upper band, as indicated) in wild-type *Tmem67^+/+^* cells. Loading control is for β-actin.

ROR2 is known to mediate non-canonical Wnt5a signalling (Mikels et al., 2009). Next, we, therefore, investigated the possible functional interactions between ROR2 and TMEM67. Endogenous ROR2 colocalised with both TMEM67 and RPGRIP1L, a marker of the transition zone (Arts et al., 2007), in ciliated mIMCD3 cells (Fig. 5A). Consistent with this observation, exogenously expressed FLAG-tagged ROR2 also partially colocalised with endogenous ROR2 and TMEM67, and in ciliated mIMCD3 cells (supplementary material Fig. S3A), and with γ-tubulin at the base of primary cilia in *Tmem67^+/+^* wild-type and *Tmem67^−/−^* mutant MEFs (supplementary material Fig. S3B). Co-immunoprecipitation experiments demonstrated that exogenous full-length and endogenous TMEM67 interacted with FLAG-tagged ROR2 (Fig. 5C,D) but not a tagged irrelevant protein (MCPH1). We then confirmed non-canonical Wnt pathway dysregulation in the absence of TMEM67 by transfecting MEFs with FLAG-ROR2. As expected, levels of the activated phosphorylated ROR2 isoform were significantly increased following treatment of wild-type *Tmem67^+/+^* MEFs with Wnt5a, but activation of ROR2 was completely abolished in the mutant *Tmem67^−/−^* cells (Fig. 5E).

### Defective branching morphogenesis in response to Wnt5a stimulation in the *Tmem67^−/−^* embryonic lung is rescued by the RhoA activator calpeptin

**Fig. 6.**
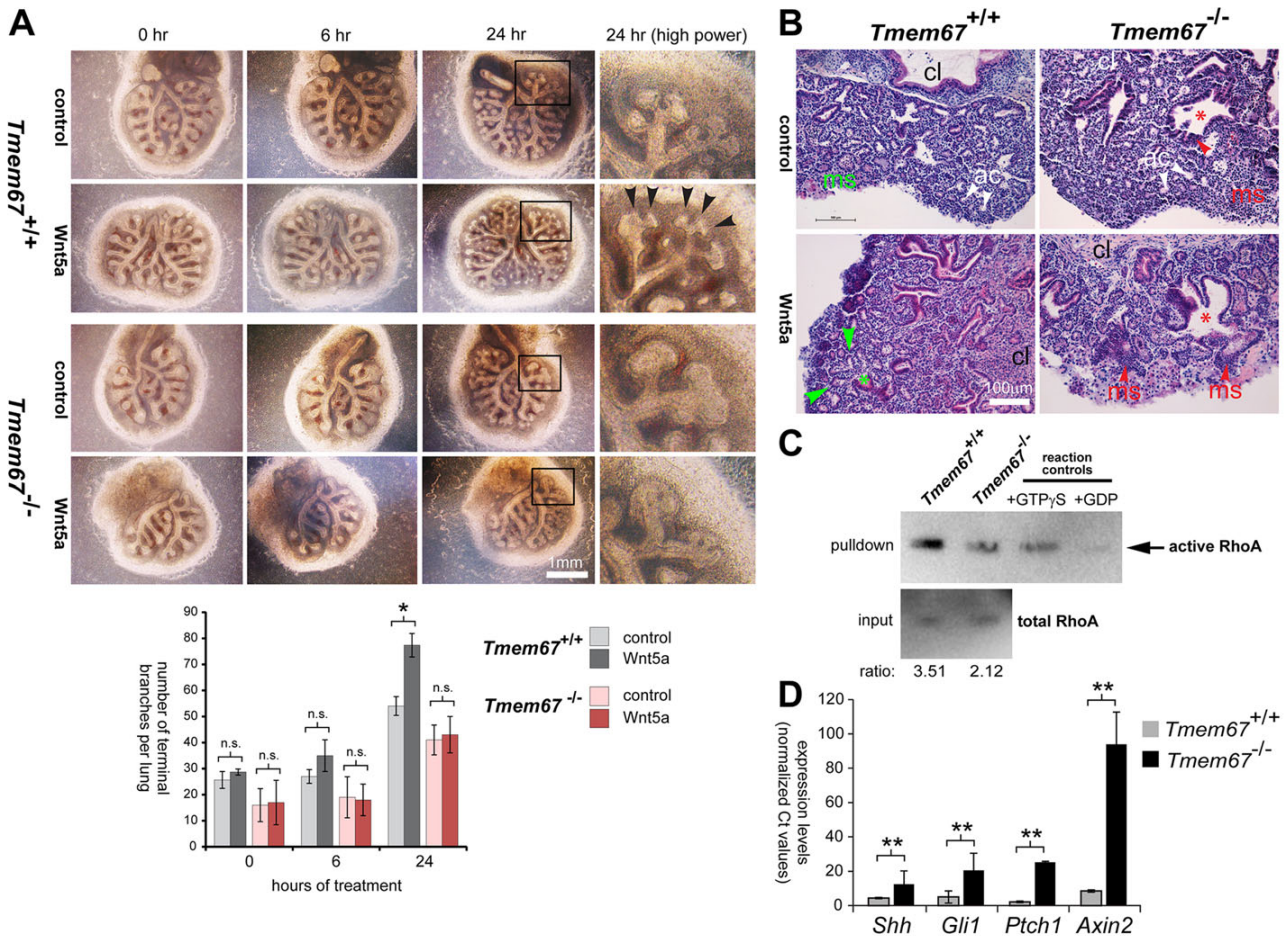
**Loss of Wnt5a-induced branching morphogenesis during *Tmem67^−/−^* embryonic lung *ex vivo* organogenesis.** (A) Embryonic (E12.5) lungs were explanted and treated for 0, 6 and 24 h with either control-conditioned medium or medium containing Wnt5a. Magnified insets (black frames) under high power are shown for 24-h treatments. Epithelial branching is significantly induced by Wnt5a in *Tmem67^+/+^* lungs, but this response is absent in *Tmem67^−/−^* lungs. The bar graph shows quantification of the total number of branches in one lung for each genotype. Values shown are means of three independent replicates and error bars indicate ±s.e.m. The statistical significance of the pair-wise comparisons are represented as *P<0.05 and n.s. for non-significant, Student's two-tailed *t*-test. (B) H&E staining of *ex*-*vivo*-cultured embryonic lung sections, showing normal acini (ac) and mesenchymal tissue (ms, in green) for wild-type *Tmem67^+/+^* lung, and the stimulation of normal epithelial branching by Wnt5a (green asterisk and arrowheads). In contrast, *Tmem67^−/−^* lungs have abnormal mesenchymal cell condensates (red arrowheads), suggesting defective epithelial-mesenchymal induction. The red asterisks indicate abnormal bronchiolar formation; cl indicates the direction of the central lung. (C) Rho activation pull-down assays of whole-cell extracts from wild-type *Tmem67^+/+^* and mutant *Tmem67^−/−^* embryonic (E15.5) lungs. Total RhoA in input material is shown as the loading control, with the ratio indicating active:total RhoA levels. A positive control for the assay (+GTPγS; loading with non-hydrolyzable GTPγS) and a negative control (+GDP; loading with GDP) are also shown. (D) Quantitative real-time PCR assays of transcript expression levels in wild-type *Tmem67^+/+^* and mutant *Tmem67^−/−^* embryonic (E15.5) lungs for *Shh*, downstream effectors of the Shh signalling pathway (*Gli1* and *Ptch1*) and a downstream effector of the canonical Wnt signalling pathway (*Axin2*). Levels of transcripts were all significantly increased in *Tmem67^−/−^* embryonic lungs, with the indicated pair-wise comparisons represented as **P<0.01, Student's two-tailed *t*-test for *n*=3 independent assays. Error bars indicate ±s.e.m.

We reasoned that, if TMEM67 is a potential receptor that directly binds to Wnt5a, absence of this receptor in the mutant would abolish or reduce responses to this ligand. We, therefore, next used an *ex vivo* organogenesis assay to follow epithelial branching morphogenesis in embryonic (E12.5) lung in response to Wnt5a. As expected, wild-type *Tmem67^+/+^* lung strongly responded to this Wnt ligand, in comparison to control treatments, with prolific elaboration of distal branching in the developing alveoli (Fig. 6A,B, supplementary material Fig. S4). Consistent with the pulmonary phenotypes of *Tmem67^−/−^* mutant embryo (Fig. 1A,B), *Tmem67^−/−^* mutant lungs grown in *ex vivo* culture were hypoplastic with significantly reduced levels of branching (Fig. 6A,B). Mutant lungs did not respond to treatment with Wnt5a, consistent with a role for TMEM67 in binding Wnt5a during embryonic processes, such as hair cell differentiation and lung morphogenesis. Consistent with a loss of responsiveness to non-canonical Wnt signalling, we observed reduced levels of active RhoA in embryonic (E14.5) *Tmem67^−/−^* mutant lung (Fig. 6C). In contrast, expression of *Shh* and downstream effectors of the Shh pathway (*Gli1* and *Ptch1*) were significantly increased in embryonic *Tmem67^−/−^* mutant lung (Fig. 6D). Consistent with previous studies (Abdelhamed et al., 2013; Garcia-Gonzalo et al., 2011), canonical Wnt signalling, as measured by *Axin2* expression, was also increased in mutant lung (Fig. 6D).

**Fig. 7.**
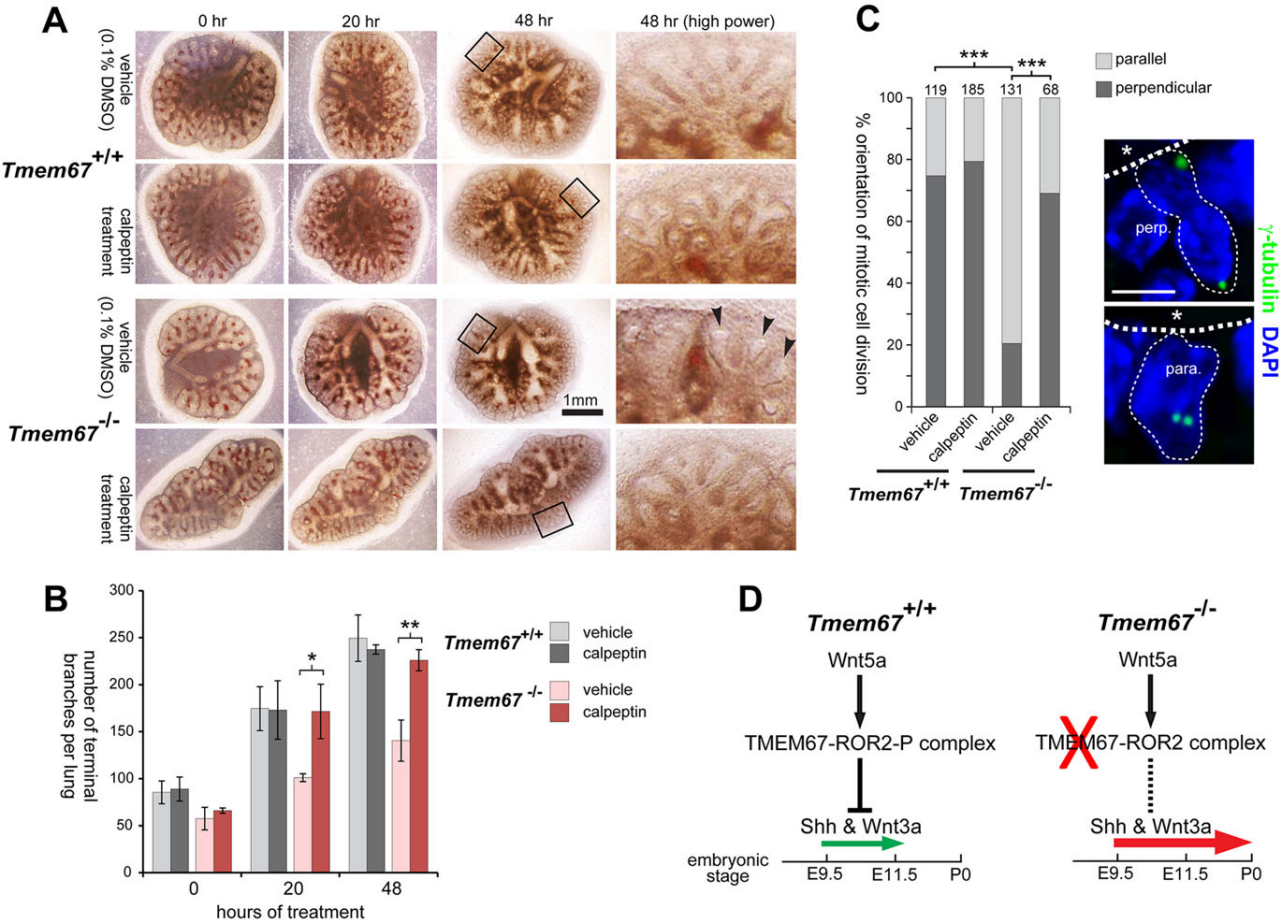
**Rescue of normal embryonic lung-branching morphogenesis and polarity in mutant *Tmem67^−/−^* tissue by *ex vivo* treatment with the RhoA activator calpeptin.** (A) Embryonic lungs (age E11.5) grown in culture for the indicated times after treatment with either vehicle control (0.1% DMSO) or calpeptin at final concentration 1 unit/ml for 3 h. *Tmem67^−/−^* lungs had abnormally dilated branches (arrowheads) surrounded by areas of condensed mesenchyme, in contrast to the fine distal branches visible in *Tmem67^+/+^* lungs. Calpeptin treatment of mutant *Tmem67^−/−^*lungs resulted in more developed branch development and a general morphology that was similar to the wild-type lungs. Magnified insets are indicated by the black frames and shown on the right. (B) The bar graph shows the quantification of the total number of terminal branches per lung (total *n*=3) for each genotype and treatment condition. The statistical significance of the indicated pair-wise comparisons is *P<0.05 and **P<0.01, Student's two-tailed *t*-test. Error bars indicate ±s.e.m. (C) The polarity of mitotic cell division is rescued by treatment with calpeptin from predominantly parallel (para.) in mutant alveoli to predominantly perpendicular (perp.) divisions, as observed in wild-type epithelia. The statistical significance of the indicated pair-wise comparisons is ***P<0.001, chi-squared test, with the total number of cells counted in ten fields of view indicated above each bar. Representative examples of mitotic divisions, visualised by γ-tubulin (green) and indicated by the fine dotted lines, are shown on the right. Apical surfaces are highlighted by the broad dotted lines, with asterisks indicating the alveolar lumen. Scale bar: 20 μm. (D) Schematic in which signalling through the Wnt5a-TMEM67-ROR2 axis normally represses Shh and canonical Wnt (Wnt3a) signalling to moderate levels (small green arrow) between embryonic ages E9.5 and E11.5. Loss or mutation of any component in this axis (red cross) causes loss of repression (dashed line) with Shh and canonical Wnt pathway de-regulation and ectopic expression of Shh at later gestation ages (large red arrow). This contributes to pulmonary hypoplasia with condensed mesenchyme and impaired development of the alveolar system in the ciliopathy disease state.

In the absence of TMEM67, ROR2 phosphorylation is, therefore, lost and the normal regulation of non-canonical Wnt signalling is disrupted. We reasoned that activation of a more downstream target of this pathway could potentially enhance lung maturation and rescue the abnormal branching, mimicking the correct responses to Wnt5a. To test this hypothesis, we used the *ex vivo* organogenesis assay to treat embryonic (E15.5) wild-type *Tmem67^+/+^* and mutant *Tmem67^−/−^* lungs with calpeptin. Calpeptin is a dipeptide aldehyde that inhibits myosin light-chain phosphorylation connected to stress-fibre formation, specifically targeting regulators of the Rho sub-family of GTPases and selectively activating RhoA (Schoenwaelder and Burridge, 1999; Schoenwaelder et al., 2000). Mutant lungs at embryonic ages E11.5 and E13.5 showed areas of delayed and abnormally dilated branches surrounded by areas of condensed mesenchyme (Fig. 7A, supplementary material Fig. S5A). Treatment with calpeptin resulted in the appearance of more developed branches and less condensed mesenchyme, closely resembling the morphology of wild-type lung at both E11.5 and E13.5 (Fig. 7A,B, supplementary material Fig. S5A). Histological assessment of these developmental changes after calpeptin treatment showed that *Tmem67^−/−^* lungs at E13.5 had a higher number of developing alveoli and showed greatly reduced mesenchymal cell condensations, with maturation comparable to wild-type lungs (supplementary material Fig. S5B). In wild-type *Tmem67^+/+^* embryonic lungs, the orientation of mitotic division in alveolar epithelial cells was predominately perpendicular to the apical cell surface and basement membrane (Fig. 7C). In mutant *Tmem67^−/−^* alveoli, mitotic divisions were predominantly parallel, but treatment with calpeptin rescued normal polarity (Fig. 6C).

## DISCUSSION

We have previously described the severe multi-organ developmental defects in the *B6;129P2-Tmem67^tm1Dgen/H^* knockout mouse that reiterate the clinical features of MKS and JBTS (Abdelhamed et al., 2013). All *Tmem67^−/−^* mutants that were examined, developed incomplete laterality defects that manifested in late gestation as left lung isomerism (Fig. 1A) and were occasionally associated with dextrocardia (Fig. 1E,F). Pulmonary hypoplasia was a consistent finding in the *Tmem67^−/−^* embryos and pups (Fig. 1A,B), although this is frequently under-reported in human ciliopathies and not considered an essential diagnostic clinical feature of MKS in humans (Salonen, 1984). However, it has been reported recently that, for MKS, death occurs *in utero* or within hours after birth because of the pulmonary hypoplasia, which can be considered as the leading cause of death in human MKS patients (Roy and Pal, 2013).

Previously, we have shown that TMEM67 is required for epithelial branching morphogenesis in three-dimensional *in vitro* tissue culture (Dawe et al., 2007). The present study now provides the first evidence that TMEM67 is essential for correct *in vivo* branching morphogenesis in lung alveolar system development (Fig. 6A,B). The similarity in the overall cardiopulmonary phenotypes (Oishi et al., 2003) and the biliary developmental malformations (Kiyohashi et al., 2013) for *Wnt5a*,* Ror2* and *Tmem67* knockout mice (Fig. 1) strongly suggests that TMEM67 mediates signalling by either the Wnt5a ligand or the ROR2 co-receptor. A marked phenotype of *Wnt5a^−/−^* mice is convergent-extension defects with misorientation of ohc and ihc stereociliary bundles (Qian et al., 2007). To further test whether Wnt5a signals through TMEM67 we, therefore, investigated the morphogenesis of the cochlea in neonatal *Tmem67^−/−^* mice.

In the present study, we now show that TMEM67 is a key regulator of cilium-dependent stereociliary hair-bundle orientation. In *Tmem67* mutant mice, ohc had misoriented hair bundles (Fig. 2E) with an apparent physical dissociation of the basal body/kinocilium complex from the hair bundle (Fig. 2F,G). This uncoupling may arise from aberrant migration of the basal body, during a period of embryonic development immediately prior to the initial growth of the stereocilia (supplementary material Fig. S2). In mutant ihc, the basal body migrated towards the abneural pole of the cell, but the fine control of its final positioning appeared to be variable. These results are consistent with our previous work, which implied that TMEM67 is mediating centriole migration to the apical membrane of polarised cells with the consequent formation of a primary cilium (Dawe et al., 2007). TMEM67 also contributed to ciliogenesis in the organ of Corti, although this appeared to be specific to the non-sensory supporting cells because all sensory hair cells were ciliated. This observation is consistent with previous results in ciliated cell lines (Dawe et al., 2007), in other tissues of *Tmem67^−/−^* mutants (Adams et al., 2012; Abdelhamed et al., 2013), and in the organ of Corti of the *bpck* mouse (Leightner et al., 2013). The *bpck* mouse carries a 245-kb deletion that includes the *Tmem67* gene, and is therefore a null mutant (Cook et al., 2009). Leightner et al. (2013) also reported stereociliary alignment and ciliogenesis defects in *bpck* mutant neonates, but did not investigate basal body migration or positioning defects in embryos (Leightner et al., 2013).

The defects of hair-bundle orientation in both *bpck* and *Tmem67^−/−^* lines are similar to those observed in mouse models of the human ciliopathies, such as the Alström syndrome (Jagger et al., 2011), BBS (May-Simera et al., 2009; Ross et al., 2005), and the *Kif3a* ciliary mutant (Sipe and Lu, 2011). Unlike *Kif3a^−/−^* mice, however, *Tmem67* mutants had the expected number of hair cell rows and the length of the sensory epithelium was comparable to that in controls (Fig. 2B,E), and both PCP and apical planar asymmetry were normal (Fig. 3) indicating that cochlear convergent-extension mechanisms were unaffected by loss of TMEM67. In *Kif3a^−/−^* hair cells, basal body position shows little correlation with the hair-bundle orientation (Sipe and Lu, 2011), comparable to the orientation defects observed in *Tmem67* mutants (Fig. 2E,G), suggesting that hair-bundle orientation does not necessarily predict the position of the basal body (supplementary material Figs S1 and S2B). The basal body, therefore, appears to be a better assay of the PCP axis (Sipe and Lu, 2011). Importantly, the *Tmem67* model system also provides *in vivo* confirmation of previous *in vitro* studies that suggested an essential role of TMEM67 in mediating centriolar migration to the apical membrane during cell polarisation (Dawe et al., 2007).

Our biochemical data also suggest that non-canonical Wnt signalling by Wnt5a is mediated or regulated, at least in part, by TMEM67 through a ciliary-dependent mechanism. In *ex-vivo*-cultured *Tmem67^−/−^* lungs, a reduction in the number of epithelial branches was detected from E12.5 (Fig. 6A). Wnt5a treatment failed to induce an increase in epithelial branching in *Tmem67^−/−^* lungs, whereas wild-type lungs responded to this treatment with prolific branching morphogenesis (Fig. 6A, supplementary material Fig. S4), suggesting that *Tmem67^−/−^* lungs are unresponsive to non-canonical Wnt5a stimulation. A proposed functional interaction between Wnt5a, ROR2 and TMEM67 is supported by several lines of experimental evidence: preferential *in vitro* binding of the TMEM67 CRD domain to Wnt5a (Fig. 4E), the colocalisation and interaction of ROR2 with TMEM67 at the ciliary transition zone (Fig. 5A-C), and the failure of *Tmem67^−/−^* cells to phosphorylate ROR2 upon Wnt5a stimulation (Fig. 5D).

ROR2 is a member of the receptor tyrosine kinase (RTK) superfamily and the cytoplasmic regions of the RTKs family contain conserved tyrosine kinase domains (Robinson et al., 2000; Sossin, 2006; Green et al., 2008). Similar to other RTKs, ROR2 forms homodimers at the cell membrane, an event essential for receptor trans-autophosphorylation and subsequent pathway activation (Green et al., 2008; Kani et al., 2004). Wnt5a stimulation has been shown to enhance the tyrosine kinase activity of ROR2 (Liu et al., 2007, 2008; Akbarzadeh et al., 2008). Our data confirm previous reports that ROR2 phosphorylation is induced by Wnt5a only and not by Wnt3a (Fig. 5E). The loss of correct ROR2 phosphorylation upon Wnt5a stimulation in *Tmem67^−/−^* cells (Fig. 5E), therefore, suggests that TMEM67 is essential for the initiation of phosphorylation, possibly by mediating homodimerisation. TMEM67, therefore, appears to be a receptor of non-canonical Wnt signalling that, preferentially, binds Wnt5a with the extracellular cysteine-rich domain (CRD) and mediates downstream signalling through ROR2 as a co-receptor.

In our present report, we describe that lung hypoplasia in *Tmem67^−/−^* depends on non-canonical Wnt signalling downstream of Wnt5a/ROR2, for which TMEM67 appeared to be essential for signalling responses in the developing lung (Fig. 6). This is consistent with the previous finding that non-canonical Wnt5a signalling is essential for proper lung development through controlling epithelial branching (Li et al., 2002). Defects in lung-branching morphogenesis and the orientation of mitotic divisions in *Tmem67^−/−^ ex*-*vivo*-cultured lungs were rescued by treatment with the RhoA activator calpeptin (Fig. 7A-C, supplementary material Fig. S5). This confirms previous reports that RhoA activation is essential for accelerated branching in the developing lungs (Moore et al., 2002, 2005; Cloutier et al., 2010).

Non-canonical Wnt signalling downstream of Wnt5a was downregulated in *Tmem67^−/−^* lungs (Fig. 6C). However, this was accompanied by increased expression of *Shh* transcripts as well as downstream effectors of both the Shh pathway (*Gli1* and *Ptch1*) and canonical Wnt signalling (*Axin2*; Fig. 6D), indicating upregulation of both the canonical Wnt and the Shh pathways. This is consistent with a previous report, which describes Wnt5a signalling as essential for inhibition of *Shh* signalling in the developing lungs after mid-gestation (Li et al., 2005). This might also explain the greater deregulation of Wnt signalling compared with Shh signalling at the mid-gestation time point (E15.5) that we assayed for transcript expression (Fig. 6D). For *Tmem67^−/−^* mice we, therefore, suggest that the loss of TMEM67 prevents Wnt5a-mediated inhibition of *Shh* signalling in the mutant lungs (Fig. 7D). Interestingly, a pulmonary phenotype similar to that of *Tmem67^−/−^* is observed after ectopic overexpression of *Shh* in the developing murine lung after mid-gestation periods (Bellusci et al., 1997). Increased *Axin2* expression in *Tmem67^−/−^* mutant lungs could similarly be explained by the lack of any inhibitory effect of the non-canonical Wnt5a ligand on canonical Wnts, because both functional classes of Wnt have previously been shown to competitively inhibit binding to their receptor site (Grumolato et al., 2010). This model is also consistent with our previous *in vitro* results in *Tmem67^−/−^* cells (Abdelhamed et al., 2013). We, therefore, propose a model in which signalling through the Wnt5a-TMEM67-ROR2 axis normally represses both Shh and canonical Wnt signalling (Fig. 7D). Loss or mutation of any component in this axis causes deregulation of Shh and canonical Wnt signalling as well as ectopic expression of Shh and Wnt, contributing to the pulmonary hypoplasia, condensed mesenchyme and impaired development of the alveolar system observed in the ciliopathy disease state. Targeting the Wnt5a-TMEM67-ROR2 signalling axis downstream of the receptor site could, therefore, provide a potential basis for therapeutic intervention to reduce or prevent lung hypoplasia in ciliopathies.

## MATERIALS AND METHODS

### Ethics statement

Animal studies described in this paper were carried out under the guidance issued by the Medical Research Council in *Responsibility in the Use of Animals for Medical Research* (July 1993) in accordance with UK Home Office regulations under the Project Licence no. PPL40/3349.

### Animals

B6;129P2-Tmem67^tm1Dgen/H^ heterozygous knockout mice were derived from a line generated by Deltagen Inc. (San Mateo, CA, USA) and made available from MRC Harwell through the European Mutant Mouse Archive (see website https://www.infrafrontier.eu/ strain number EM:02370). The targeting *β-Gal-neo* (*geo*) construct inserts downstream of exon 1 of the *Tmem67* gene (Abdelhamed et al., 2013). Genotyping was done by PCR on DNA extracted from tail tips or the yolk sac of E11.5-E15.5 embryos, or ear biopsies of adult mice.

### Cells

Human embryonic kidney (HEK293) and mouse inner medullary collecting duct (mIMCD3) cells were grown in Dulbecco's modified Eagle's medium (DMEM)/Ham's F12 supplemented with 10% foetal calf serum at 37°C/5% CO_2_, essentially as described previously (Abdelhamed et al., 2013). The derivation and culture of mouse embryonic fibroblasts (MEFs) has been described previously (Adams et al., 2012) MEFs were grown in DMEM/Ham's F12 supplemented with 10% foetal calf serum and 1% penicillin-streptomycin at 37°C/5% CO_2_.

### Cloning, plasmid constructs and transfections

Full-length human *TMEM67/MKS3* was cloned into the pCMV-HA vector as described previously (Adams et al., 2012). The pSec2A-TMEM67-Nt construct (encoding amino acids F39-T478, and including the cysteine-rich domain and β-sheet motifs, Fig. 4A) was constructed by standard sub-cloning of a PCR product containing *Hind*III and *Not*I restriction sites after amplification with Platinum *Taq* DNA Polymerase High Fidelity (Life Technologies Ltd, Paisley, UK). Inserts were verified by bidirectional DNA sequencing. Missense mutations were introduced using the QuickChange mutagenesis kit (Stratagene Inc., La Jolla, CA, USA) and verified by DNA sequencing. Plasmid pEF1a-mROR2WT (Mikels et al., 2009) was obtained from Addgene, Cambridge, MA, USA (plasmid number 22613). For transfection with plasmids, cells at 80% confluency were transfected by using Lipofectamine 2000 (Life Technologies Ltd) according to the manufacturer's instructions and as described previously (Dawe et al., 2009).

### Antibodies and fluorescent markers

The following primary antibodies were used: mouse anti-β-actin (clone AC-15; Abcam Ltd, Cambridge, UK); mouse anti-Ki67 (Merck Millipore Inc., Feltham, UK); mouse anti-FLAG (clone M2; Sigma-Aldrich Co. Ltd, Gillingham, UK); rabbit polyclonal anti-Vangl2 (1:500; a kind gift from Mireille Montcouquiol, INSERM Université Bordeaux, France); rabbit polyclonal anti-Gαi3 (1:400; G4040, Sigma-Aldrich); rabbit polyclonal anti-atypical protein kinase C (PKC-ζ; 1:400; sc216, Santa Cruz); goat anti-ROR2 (R&D Systems Inc., Minneapolis, MN, USA); guinea pig anti-RPGRIP1L (SNC040) polyclonal antibody at 1:200 (Arts et al., 2007), a kind gift from Ronald Roepman, Radboud UMC, Nijmegen, The Netherlands; and rabbit anti-TMEM67 C-terminus polyclonal antibody at 1:100 (Abdelhamed et al., 2013). Microtubules were stained with mouse monoclonal antibody against acetylated α-tubulin (clone 6-11B-1; Sigma-Aldrich Co. Ltd; 1:1000), shown previously to detect cochlear ciliary axonemes (Jagger et al., 2011; May-Simera et al., 2009). Ciliary basal bodies were immunolocalised by using a rabbit polyclonal anti-ALMS1 antibody at 1:200 (Jagger et al., 2011). F-actin was stained with tetramethyl-rhodamine (TRITC)-conjugated phalloidin (Sigma-Aldrich Co. Ltd) at 1:1000. Secondary antibodies were Alexa-Fluor-568-conjugated goat anti-mouse IgG, Alexa-Fluo-r488-conjugated goat anti-rabbit IgG, Alexa-Fluor-568-conjugated goat anti-guinea-pig IgG, Alexa-Fluor-633-conjugated goat anti-rabbit IgG and Alexa-Fluor-488-conjugated donkey anti-goat IgG (Life Technologies Ltd).

### Preparation of tissue sections, histology and immunohistochemistry

Mouse embryos or dissected tissues were fixed in 4% (w/v) paraformaldehyde (PFA)and embedded in paraffin wax. Thin sections (4 μm) were cut onto Superfrost Plus slides (VWR International Ltd, Lutterworth, UK) and were deparaffinised and rehydrated using standard methods. Sections were stained with haematoxylin and eosin (VWR International Ltd) for 2 min, then dehydrated in ethanol, cleared in xylene and mounted in DPX. For immunohistochemistry, tissue sections were deparaffinised and rehydrated. Epitope recovery was obtained by boiling in 1 mM EDTA pH 8.0, for 2 min using pressure cooker, followed by 20 min cooling. Blocking and application of primary antibodies was as described (Dawe et al., 2007). Appropriate HRP-conjugated secondary antibodies (Dako UK Ltd, Ely, UK) were used (final dilutions of ×10,000-25,000). Sections were developed in Sigma Fast 3,3′-diaminobenzidine (DAB) with CoCl_2_ enhancer and counterstained with Mayer's haematoxylin (Sigma-Aldrich Co. Ltd).

### Cochlear immunofluorescence and confocal microscopy

For TMEM67 immunofluorescence experiments cochleae were fixed using 2% PFA in phosphate-buffered saline (PBS) for 20 min at room temperature. For morphogenesis studies cochleae were fixed using 4% PFA in PBS overnight at 4°C. The organs of Corti were dissected, and divided lengthwise two or three times for subsequent mounting. Tissues were permeabilised and blocked (0.1% Triton-X 100 with 10% normal goat serum in PBS) for 30 min at room temperature, and then incubated in primary antibodies overnight at 4°C. Following several washes with PBS, tissues were incubated with secondary antibodies (Alexa-Fluor-568-conjugated goat anti-mouse IgG, Alexa-Fluor-488-conjugated goat anti-rabbit IgG and Alexa-Fluor-488-conjugated goat anti-mouse IgG; Life Technologies Ltd) in the dark for 30 min at room temperature. Cells or tissues were mounted on glass slides using Vectashield with diamidino-2-phenylindole (DAPI; Vector Laboratories Ltd, Peterborough, UK). Imaging was carried out using a laser scanning confocal microscope (LSM510; Carl Zeiss Microscopy GmbH, Jena, Germany) or a Nikon Eclipse TE2000-E system, controlled and processed by EZ-C1 3.50 (Nikon UK Ltd, Kingston-upon-Thames, UK) software. Images were assembled using Adobe Illustrator CS4 (Adobe Systems Inc., San Jose, CA, USA).

### Preparation of whole-cell extracts, western immunoblotting and RhoA activation assays

Whole-cell extracts (WCE) containing total soluble proteins were prepared from confluent untransfected HEK293 or IMCD3 cells, or cells that had been transiently transfected with 1.0 μg plasmid constructs in 90 mm tissue-culture dishes, or scaled down as appropriate. Ten µg of WCE total soluble protein was analysed by using SDS-PAGE (using 4-12% polyacrylamide gradient gels) and western blotting according to standard protocols using either rabbit polyclonal antisera (final dilutions of 1:200-1000) or mAbs (1:1000-5000). Appropriate HRP-conjugated secondary antibodies (Dako UK Ltd) were used (final dilutions of 1:10,000-25,000) for detection by the enhanced chemiluminescence Femto West western blotting detection system (Thermo Fisher Scientific Inc., Rockford, IL, USA) and visualised using a ChemiDoc MP imaging system (Bio-Rad Inc., Hercules, CA, USA). The activated GTP-bound isoform of RhoA was specifically assayed in pull-down assays by using a GST fusion protein of the Rho effector rhotekin (Cytoskeleton Inc., Denver, CO, USA), under conditions recommended by the manufacturer. WCEs were processed as rapidly as possible at 4°C, and snap-frozen in liquid nitrogen. Total RhoA (in input WCEs) and pull-down protein was immunodetected on western blots using a proprietary anti-RhoA monoclonal antibody (Cytoskeleton Inc.). Immunoblotting of total RhoA was used as the loading control. Ratios of active RhoA:total RhoA were calculated by quantifying band intensity using ImageLab 5.2.1 software (Bio-Rad Inc.).

### Canonical Wnt activity (TOPFlash) luciferase assays

For luciferase assays of canonical Wnt activity, we grew mouse embryonic fibroblasts in 12-well plates and co-transfected with 0.5 μg TOPFlash firefly luciferase construct (or FOPFlash, as a negative control); 0.5 μg of expression constructs (pCMV-HA-TMEM67, or empty pCMV-HA or pCMV-Myc vector); and 0.05 μg of pRL-TK (Promega Corp., Madison, WI, USA); *Renilla* luciferase construct used as an internal control reporter). Cells were treated with Wnt3a- or Wnt5a-conditioned media to stimulate or inhibit the canonical Wnt pathway. Wnt3a- or Wnt5a-conditioned media were obtained from L cells stably transfected with Wnt3a or Wnt5a expression vectors and used as described previously (Willert et al., 2003). Control medium was from untransfected L cells. Activities from firefly and *Renilla* luciferases were assayed using the Dual-Luciferase Reporter Assay system (Promega Corp.) on a Mithras LB940 (Berthold Technologies, Bad Wildbad, Germany) luminometer. Minimal responses were noticed with coexpression of the FOPFlash negative-control reporter construct. Raw readings were normalised against values from *Renilla* luciferase. Results reported are from at least four independent biological replicates.

### Protein expression and *in vitro* binding assay

Purified recombinant Wnt3a and Wnt5a proteins (R&D Systems Inc.) and purified BSA as a negative control (Sigma-Aldrich Co. Ltd), were labelled with NHS-fluorescein (Thermo Fisher Scientific Inc.), as described by the manufacturer. Unincorporated fluorescein was removed by fluorescent-dye-removal columns (Thermo Fisher Scientific Inc.) TMEM67-Nt protein (encoding amino acids F39-T478, predicted molecular mass 48 kDa) was expressed following transfection of HEK293 cells with pSec2A constructs (Life Technologies Ltd) using conditions recommended by the manufacturer. TMEM67-Nt proteins were diluted in 100 mM bicarbonate/carbonate buffer pH 9.6 and applied to Immunosorb 96-well plates (Thermo Fisher Scientific Inc.) overnight at 4°C, washed with 1× PBS, and blocked with 5% [w/v] non-fat dried milk in 1× PBS for 2 h at room temperature. Fluorescence-labelled proteins in blocking buffer were applied to plate wells, incubated for 2 h at room temperature, and then washed extensively with 1× PBS. Fluorescence retained on plates was then detected with a Mithras LB940 (Berthold Technologies) fluorimeter.

### Embryonic lung *ex vivo* culture

Embryonic E12.5 lungs were micro-dissected into cold HBSS (Life Technologies Ltd) under completely aseptic conditions. Lungs were washed in serum-free medium and transferred to a semipermeable transparent Transwell membrane with 0.4 µm pore size (Merck Millipore Inc.). The insert was placed over 1 ml of serum-free DMEMF12 medium, supplemented with penicillin, streptomycin and ascorbic acid (0.2 mg/ml) in a twelve-well plate.

### Quantitative real-time PCR (qRT-PCR)

qRT-PCR reactions were performed as described previously (Abdelhamed et al., 2013). Primer sequences are available upon request. The average Ct values of the samples were normalised to values for β-actin. Fold-difference in expression of the different genes in the mutant embryos was calculated relative to their expression in wild-type or heterozygous littermates using the standard curve method.

### Measurements and statistical analyses

Length and orientation measurements were carried out using LSM510 Image Browser 4.2 software (Carl Zeiss Microscopy GmbH). Normal distribution of data was confirmed using the Kolmogorov–Smirnov test (GraphPad Prism, GraphPad Software Inc., La Jolla, CA, USA). Pairwise comparisons were analysed with Student's two-tailed *t*-test using InStat (GraphPad Software Inc). Results reported are from at least three independent biological replicates.

## Supplementary Material

Supplementary Material
