# Supplementary material for: The Meckel-Gruber syndrome protein TMEM67 controls basal body positioning and epithelial branching morphogenesis in mice via the non-canonical Wnt pathway
Source: Dis Model Mech. 2015 Jun 1;8(6):527–41. doi: 10.1242/dmm.019083 (PMC4457033; doi:10.1242/dmm.019083)
Supplement: Supplementary Material [file supp_8_6_527__index.html]

The Meckel-Gruber syndrome protein TMEM67 controls basal body positioning and epithelial branching morphogenesis in mice via the non-canonical Wnt pathway — Supplementary Material 

# The Meckel-Gruber syndrome protein TMEM67 controls basal body positioning and epithelial branching morphogenesis in mice via the non-canonical Wnt pathway

## DMM019083 Supplementary Material

**Files in this Data Supplement:**

- **Supplementary Material**
