# Supplementary material for: The Meckel-Gruber syndrome protein TMEM67 controls basal body positioning and epithelial branching morphogenesis in mice via the non-canonical Wnt pathway
Source: Dis Model Mech. 2015 Jun 1;8(6):527–41. doi: 10.1242/dmm.019083 (PMC4457033; doi:10.1242/dmm.019083)
Supplement: Supplementary Material [file supp_8.6.527_DMM019083.pdf]

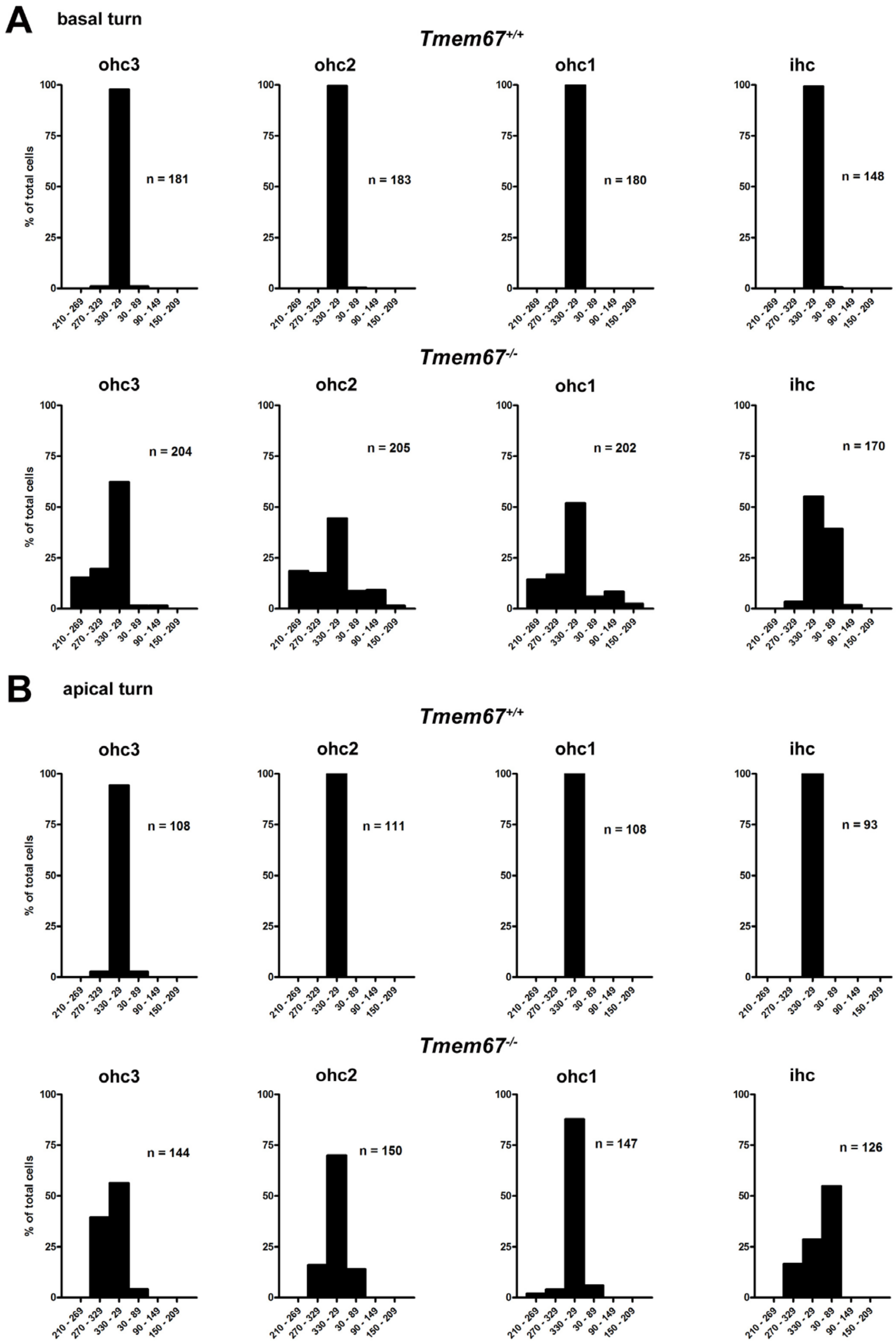

Supplementary Figure 1. Distribution of aberrant basal body position in neonatal *Tmem67$^{-/-}$* hair cells. (**A**) Basal body positions were measured (relative to the abneural pole at 0°), and binned into 6 sectors spanning 60°. In the basal cochlear turn (corresponding to ~10-20% cochlear length) of P0 *Tmem67$^{+/+}$* mice (upper panel) hair cells in all rows had basal bodies positioned very close to the abneural pole. In *Tmem67$^{-/-}$* hair cells (lower panel) there was a wider distribution of basal body position, though the abneural sector was still the most frequently occupied. (**B**) Similar differences in distribution were seen in the apical turn (~70-80%), though ihc basal bodies were most frequently located in the 30-89° sector. Data was pooled from three animals of each genotype.

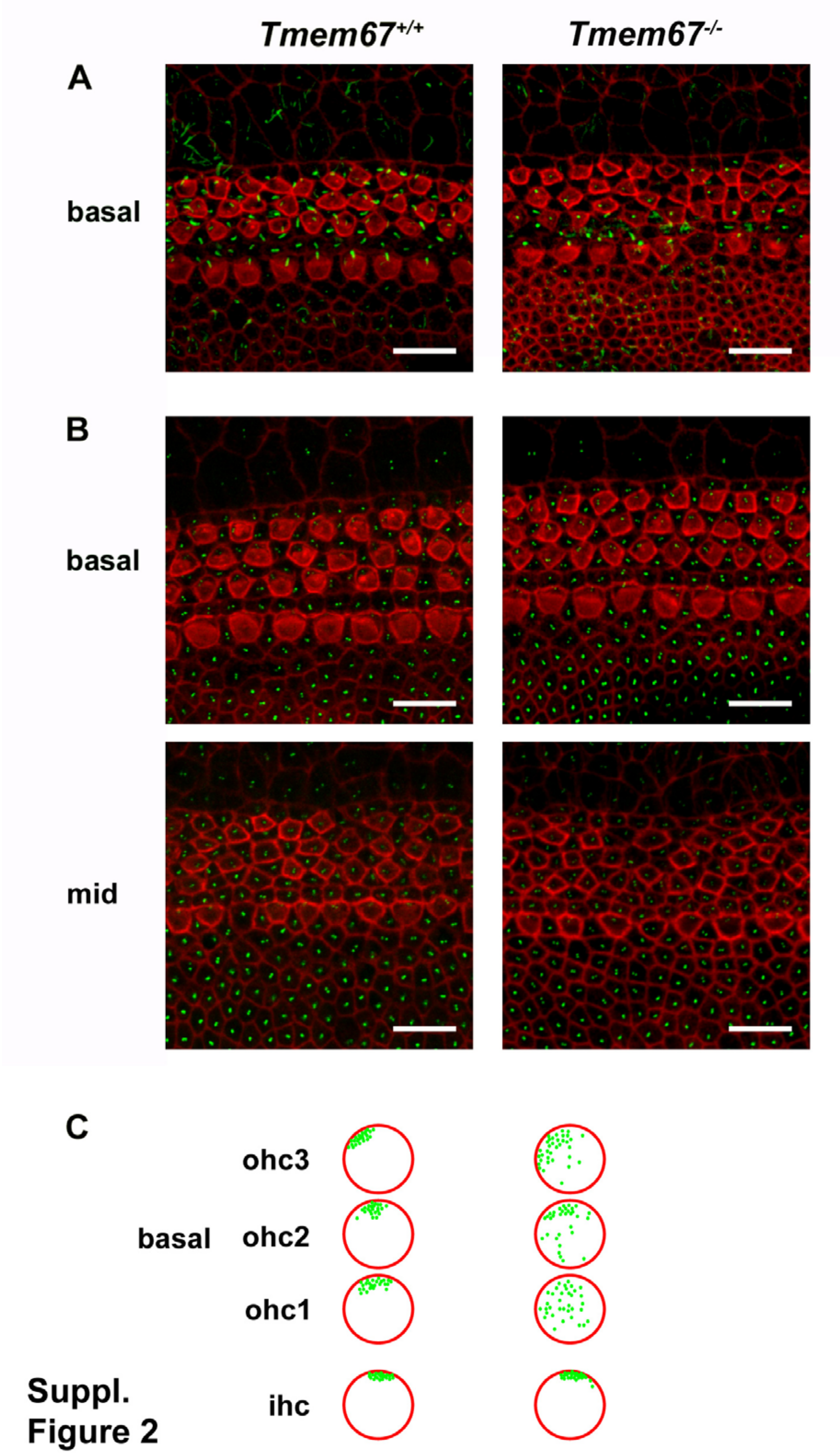

Supplementary Figure 2. Planar polarization defects during embryonic development of hair cells. (A) Wholemount preparations of basal turn organ of Corti (corresponding to ~10% cochlear length) from an E15.5 *Tmem67*$^{+/+}$ mouse (left) and a *Tmem67*$^{-/-}$ littermate (right), stained with fluorescent phalloidin (red) and anti-acetylated α-tubulin antibody (green). All *Tmem67*$^{+/+}$ hair cells and supporting cells were ciliated, but cilia were not detected on the majority of *Tmem67*$^{-/-}$ supporting cells. **(B)** Wholemount preparations stained with fluorescent phalloidin (red) and anti-ALMS1 antibody (green). In the basal region the basal bodies in *Tmem67*$^{+/+}$ inner hair cells were localised close to the abneural pole, with little deviation from 0°. In rows 1-2 the basal bodies of outer hair cells were also localised close to the abneural pole, but in row 3 they were detected some distance away. In the mid-turn region (~50%) the rows of hair cells were demarcated clearly by phalloidin staining, but abneural polarisation of basal bodies was only evident in inner hair cells, with basal bodies being localised roughly centrally in outer hair cells. Scale bars = 10 µm. **(C)** Schematic representation of the basal body position in E15.5 basal turn outer hair cells (ohc1-3) and inner hair cells (ihc). Data pooled from age-matched littermates, 2 animals from each genotype. In *Tmem67*$^{+/+}$ hair cells the basal bodies are closely packed, reflecting a stereotyped migration. In *Tmem67*$^{-/-}$ ohc there is a large variation of basal body position. The basal bodies of *Tmem67*$^{-/-}$ ihc are relatively closely packed.

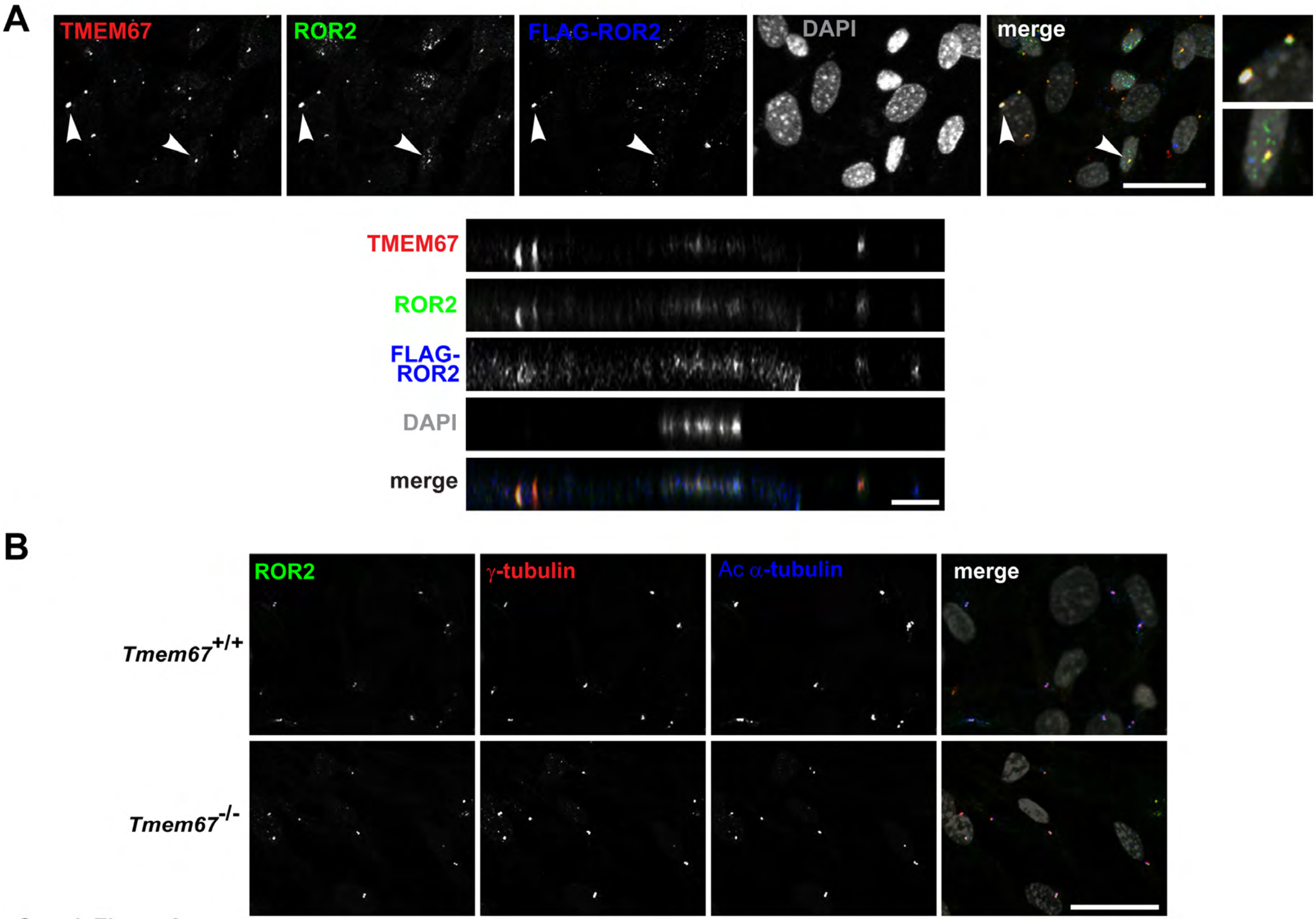

Suppl. Figure 3

Supplementary Figure 3. Localization of ROR2 to proximal regions of primary cilia. (A) Upper panels: four colour IF imaging showing that both FLAG-tagged ROR2 (blue) and endogenous ROR2 (green) co-localize with TMEM67 (red). Arrowheads indicate regions shown in magnified insets. DAPI is pseudocoloured in grey. Scale bar = 10µm. Lower panels: confocal x-z projections of FLAG-tagged ROR2, ROR2 and TMEM67 as in (A) above. Scale bar = 5µm. (**B**) Four colour IF imaging showing that endogenous ROR2 (green) partially co-localizes with g-tubulin (red) and acetylated a-tubulin (blue) at the base of the cilium in both *Tmem67*$^{+/+}$ wild-type and *Tmem67*$^{-/-}$ mutant MEFs. DAPI is pseudocoloured in grey. Scale bar = 10µm.

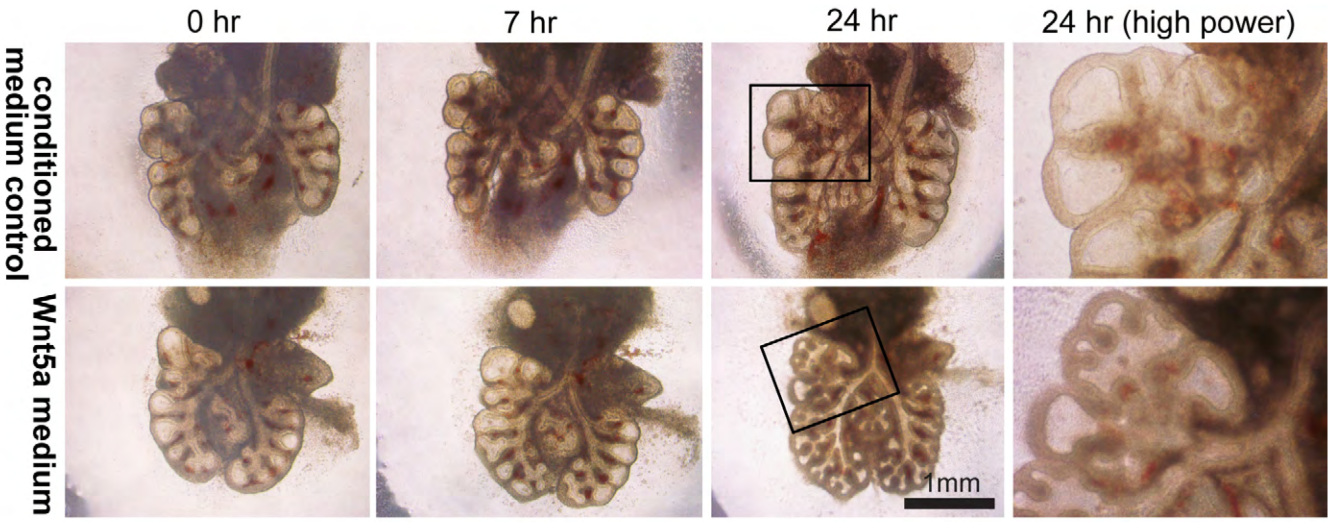

Supplementary Figure 4. Wnt5a-induced branching morphogenesis during *ex vivo* organogenesis of wild-type *Tmem67*$^{-/-}$ embryonic lung. Embryonic (E12.5) lungs were explanted and treated for 0, 7 and 24 hr with either control conditioned medium or medium containing Wnt5a. Magnified insets (black frames) under high power are shown for 24 hr treatments. Epithelial branching is significantly induced by Wnt5a in wild-type *Tmem67*$^{+/+}$ lungs.

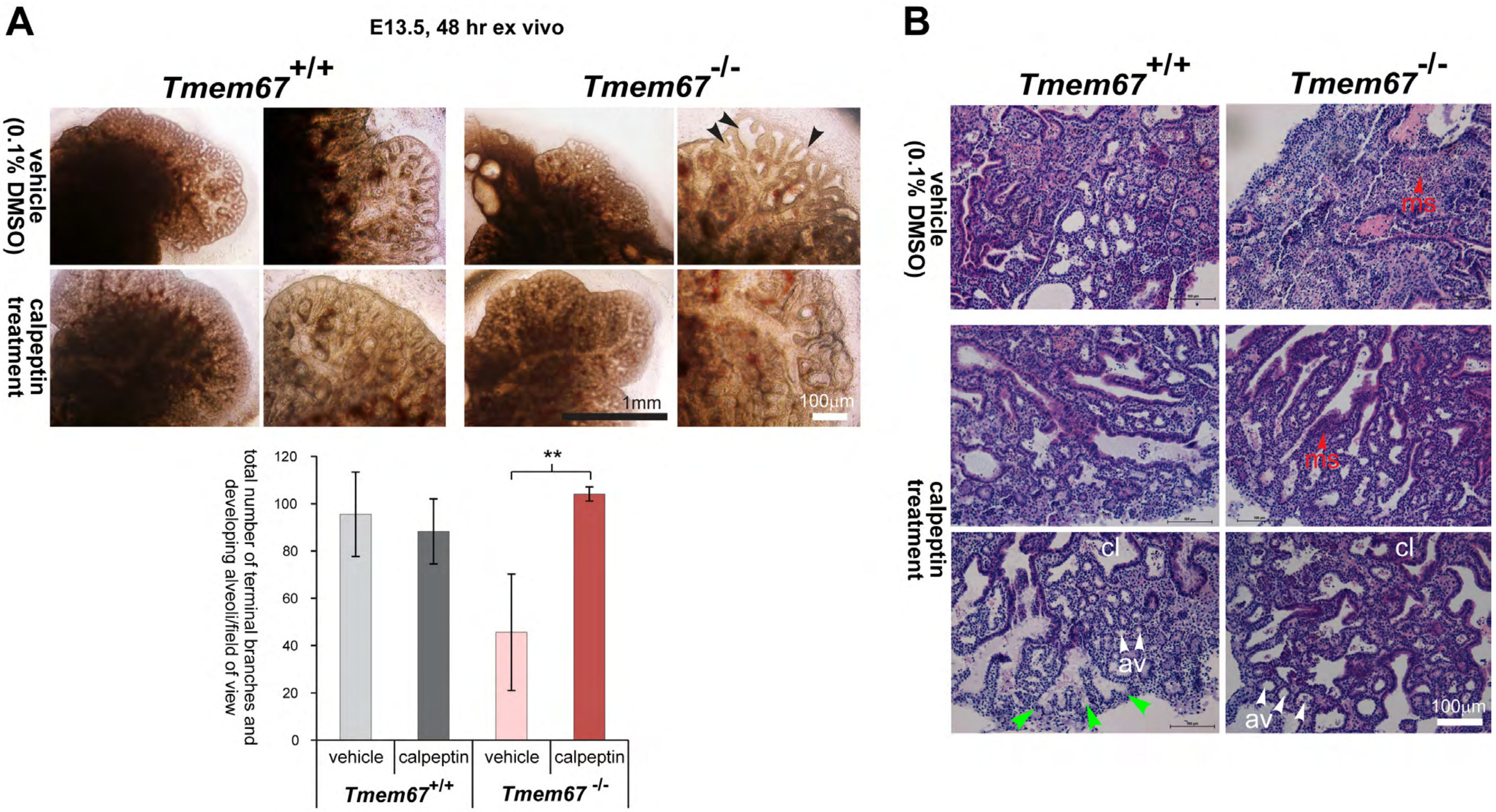

Supplementary Figure 5. Rescue of normal embryonic lung branching morphogenesis in mutant *Tmem67*$^{-/-}$ tissue by *ex vivo* treatment with the RhoA activator calpeptin. (A) Embryonic lungs at E13.5 grown in culture and treated with vehicle control (0.1% DMSO) or calpeptin at final concentration 1unit/ml for 3 hours. *Tmem67*$^{-/-}$ lungs, at this stage of development, had abnormal dilated branches (arrowheads) surrounded by areas of condensed mesenchyme. The fine distal branches that indicate further division were absent. Calpeptin treatment resulted in more normal branch development and a general morphology that was similar to the wild-type lungs. Magnified insets are shown on the right of each panel. Scale bars = 1mm, or 100µm in magnified images to the right, as indicated. The bar graph quantitates the total number of terminal branches and developing alveoli per field of view (total n=5). Indicated pair-wise comparison represented as ** for $p<0.01$, Student two-tailed t-test for n=3 independent assays. Error bars indicate s.e.m. **(B)** H&E staining of *ex vivo* cultured embryonic lung sections treated with either calpeptin or the DMSO vehicle, showing more developing alveoli (av) and less condensed mesenchymal tissue (ms, red arrowhead) for *Tmem67*$^{-/-}$ lung treated with calpeptin. *Tmem67*$^{-/-}$ lungs treated with the vehicle control appear far less developed with abnormal dilated epithelial branches, very few developing alveoli (av), and large areas of abnormal mesenchymal cell condensates (ms, red arrowhead). Normal epithelial branching in the *Tmem67*$^{+/+}$ lung is indicated (green arrowheads); cl indicates the direction of the central lung. Scale bars = 100µm.

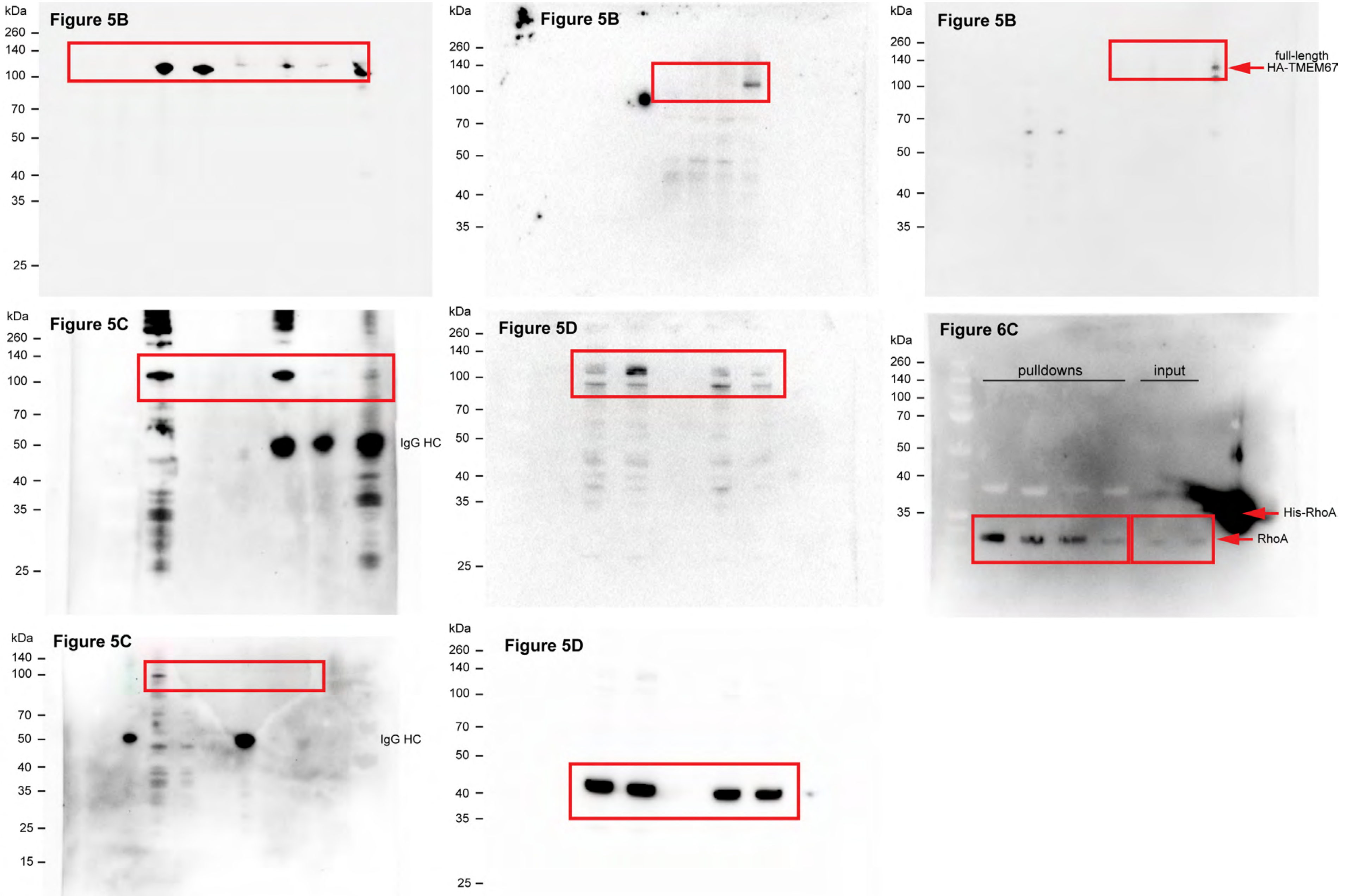

Suppl. Figure 6

Supplementary Figure 6. Original western immunoblotting images. Original, unprocessed images are shown for data presented in **Figures 5B-D & 6C**, with red frames indicating the regions used in the final figures. The molecular weight of protein size standards (kDa) are indicated to the left of all immunoblots.
